# Sustainable waste valorization through vermicomposting: optimizing rabbit manure–plant/kitchen waste mixtures for agroecosystem benefits

**DOI:** 10.1038/s41598-026-61927-3

**Published:** 2026-07-19

**Authors:** Heba G. R. Younis, Mohamed E. Abuarab, Hassan R. S. Abdellatif

**Affiliations:** Agricultural Engineering Department, Faculty of Agriculture, Cairo University, Giza, 12613 Egypt

**Keywords:** Vermicomposting, Rabbit manure, Plant and kitchen wastes, Antioxidant activity, Soil biological activity, Vegetable productivity, Ecology, Ecology, Environmental sciences, Plant sciences

## Abstract

The growth in organic waste generation from agriculture and homes and excessive use of chemical fertilizers call for sustainable nutrient management practices. We investigated the impact of vermicompost made from rabbit manure (R_m_) and plant/kitchen waste (P_w_) in five proportions (0-100%) on compost properties, vegetable growth, and soil fertility. The vermicomposting process, utilizing the earthworm species *Eisenia fetida*, spanned 75–120 days, following which it was administered to tomato, lettuce, and carrot plants in the laboratory. The physical and chemical characteristics of the compost were affected by the feedstock. Higher P_w_ resulted in lower pH, electrical conductivity (EC), and bulk density, but higher organic matter, porosity, and water retention. However, Rm-dominated treatments had greater macronutrients and micronutrients. The 50% R_m_: 50% P_w_ amendment provided an ideal C/N ratio (21), improved enzyme activity, and a diverse microbial community. All treatments significantly improved crop yields over the control, with the 50:50 mixture achieving maximum yield for tomato (10.2 kg m^−2^) and lettuce (4.5 kg m^−2^), while P_w_-rich treatments were best for carrot yield. Soil quality was significantly enhanced (structure, CEC, and microbial activity). The results show that the R_m_:P_w_ ratio affects the quality of the vermicompost and enables sustainable waste valorization and soil fertility. This research provides an R_m_:P_w_ compositional approach to link feedstock ratios to compost maturity, microbial community, crop yield, and soil properties after application. It reveals an optimal R_m_:P_w_ ratio (50:50) that optimally balances compost stability, crop performance and environmental benefits, offering a scalable approach to integrated organic waste valorization in circular food systems.

## Introduction

Globally, the exponential growth of population and industrialization has led to a critical surge in diverse and hazardous waste streams, ranging from non-biodegradable plastics to rapidly accumulating electronic waste (E-waste), posing severe environmental challenges^1,2^. Concurrently, Over the past few decades, agricultural intensification has led to a significant rise in synthetic fertilizer applications to sustain global food production. But this practice has resulted in severe environmental and agronomic problems, such as soil degradation, nutrient imbalances, water pollution and loss of biodiversity. These negative consequences have driven the need for a shift to sustainable farming practices focusing on ecological sustainability, resource conservation and long-term soil fertility^3^. In this context, organic fertilizers have found a place, with vermicompost being one of the most promising organic amendments for soil and crop improvement.

Vermicompost is a stable organic fertilizer derived from the bio-oxidative process of organic wastes by earthworms and microorganisms. The end product is a humus-like substance containing essential macro- and micronutrients, enzymes, vitamins and plant growth regulators. Besides its fertilizing effect, vermicompost is also important in organic waste management, thus it contributes to circular economy and sustainable resource use^3^. Recent research has shown that it can be used to mitigate both soil fertility loss and pollution, making it an integral part of sustainable agriculture. Vermicomposting (VC), which involves the earthworms and microorganisms, is an eco-friendly approach to dealing with the problem of organic waste, and with the added advantage of enabling the production of high-quality compost^4,5^. Vermicomposting (or worm composting) is a controlled decomposition of organic matter by earthworms. It not only results in the production of vermicompost but also reduces waste and pathogens. Vermicompost is a better organic manure compared to regular compost because of its high nitrogen content. It holds more water, enhances soil structure, stimulates microbial activity and contains more nutrients, such as nitrogen, phosphorus, potassium and other micronutrients, essential for plant growth^6,7^.

Although rapid composting technologies, such as rotary drum composting, aerated composting, and black soldier fly (BSF) larvae-based bioconversion, efficiently reduce processing time and increase waste treatment capacity, vermicomposting remains a complementary waste valorization technology because it produces a biologically active, humified, and nutrient-rich organic fertilizer enriched with beneficial microorganisms and plant growth-promoting substances. Consequently, vermicomposting prioritizes compost quality and agronomic value for sustainable crop production, whereas rapid composting technologies primarily emphasize waste stabilization efficiency and processing throughput^8–10^.

Vermicompost is increasingly recognized as a sustainable soil amendment because of its ability to improve the physical, chemical, and biological properties of soils. Physically, it enhances porosity, aggregate stability, and water-holding capacity, thereby creating favorable conditions for root development and improving soil moisture retention. Chemically, vermicompost supplies readily available nutrients and increases soil cation exchange capacity and buffering capacity, resulting in improved nutrient availability and retention. Beyond macronutrients, manure-based vermicompost can also provide essential micronutrients such as zinc (Zn) and copper (Cu), which are commonly deficient in intensively cultivated soils. Through gradual organic matter decomposition and nutrient mineralization, these micronutrients become more available to plants, while humified organic compounds may reduce nutrient fixation and enhance uptake efficiency. Zinc supports enzyme activity, protein synthesis, and plant growth regulation, whereas copper plays key roles in photosynthesis and metabolic processes. Biologically, vermicompost stimulates microbial abundance and enzymatic activity that sustain nutrient cycling and long-term soil health^11^. Collectively, these improvements contribute to sustained soil fertility and reduced dependence on synthetic fertilizers, establishing a foundation for more productive and resilient cropping systems.

These improvements in soil functionality are particularly relevant in green vegetable production, where crop performance is closely linked to soil quality and nutrient availability. Recent studies have demonstrated that vermicompost enhances seed germination, root establishment, nutrient uptake, biomass accumulation, and overall yield^12^. In addition, vermicompost positively influences plant physiological processes, including chlorophyll synthesis and photosynthetic efficiency, thereby supporting greater productivity. It may also improve tolerance to abiotic stresses such as water limitation and salinity through enhanced antioxidant responses and more efficient nutrient utilization^13^. As these physiological and agronomic benefits accumulate, improvements become evident not only in crop productivity but also in the nutritional quality of harvested vegetables.

Accordingly, vermicompost contributes to the enhancement of vegetable quality and nutritional value. Studies on leafy vegetables, including lettuce, have reported increased concentrations of nutrients, antioxidants, and bioactive phytochemicals following vermicompost application, with positive implications for human nutrition and food safety^14^. Furthermore, reduced reliance on chemical fertilizers may lower chemical residues in vegetables and support safer food production systems. These agronomic advantages extend beyond the field level and connect directly to broader environmental and sustainability outcomes.

Beyond crop production, vermicompost supports environmental sustainability through greenhouse gas mitigation, reduced nutrient runoff, and increased carbon sequestration. By transforming organic waste into value-added agricultural inputs, vermicomposting decreases pressure on landfill systems and reduces environmental pollution, reinforcing circular resource use and sustainable agricultural development^15,16^.

Among different feedstocks, rabbit manure has shown particular promise as a substrate for vermicomposting. Vermicomposting rabbit manure mixed with grass clippings improved moisture retention and optimized salinity and pH conditions, generating a product more suitable for plant growth than composts derived from other organic residues such as goat manure or rosemary pruning waste^17^. In addition, vermicomposting generally increases the concentrations of nitrogen (N), phosphorus (P), and potassium (K), producing nutrient-rich amendments with strong potential for vegetable cultivation^18–20^. Although direct evidence for rabbit manure–based vermicompost in vegetables remains limited, findings from studies on other crops suggest similar benefits. For example, media containing 10–30% vermicompost improved cucumber seedling growth and yield, while applications in corn and parsley enhanced growth performance and nutrient accumulation^19^. These findings suggest that rabbit manure–derived vermicompost may represent an effective biofertilizer for sustainable vegetable production.

Household organic wastes, including fruit and vegetable peels, may further improve vermicomposting performance when combined with rabbit manure. These materials provide carbohydrates, moisture, and degradable organic substrates that support earthworm activity and microbial processes. Integrated vermicomposting systems combining plant and animal residues have demonstrated favorable processing efficiency, improved nutrient content, and enhanced compost quality; however, optimization of feedstock proportions and processing conditions remains necessary^21^.

Despite its substantial potential, wider commercial adoption of vermicompost in vegetable production remains constrained by variability in nutrient composition associated with feedstock type, inconsistencies in product quality, and the absence of standardized production systems. Additional concerns related to pathogens, salinity, and operational management also limit large-scale implementation. Therefore, future research should focus on optimizing application rates, evaluating long-term performance across diverse agro-ecological environments, and developing standardized production protocols to facilitate broader integration into conventional agricultural systems^22^.

Overall, vermicompost represents a valuable organic fertilizer that advances sustainable and green vegetable production through improvements in soil quality, crop productivity, nutritional value, and environmental performance. By enhancing soil physicochemical properties, reducing chemical inputs, and promoting waste recycling within circular agricultural systems, vermicomposting offers a practical pathway toward more resilient and sustainable food production systems^3,12,23^.

While the benefits of using mixed organic wastes for vermicomposting are acknowledged, little work has focused on the impact of various ratios of rabbit manure and kitchen waste on the quality and effectiveness of vermicomposting. It is critical to determine how different proportions of these complementary wastes affect decomposition, nutrient composition, microbial activity, and other characteristics of compost. This information is critical for optimizing vermicomposting processes and maximizing organic waste utilization^24^.

Despite the known benefits of vermicomposting, there is a critical knowledge gap regarding the synergistic effects of combining rabbit manure (Rm) and plant-based kitchen waste (Pw) across a complete gradient, particularly concerning their subsequent performance on diverse crop categories. To address this gap, this study introduces a novel approach that systematically determines the optimal mixing ratios between these two contrasting organic streams and comprehensively tracks their multidimensional impacts from production to soil and crop yields. Specifically, this research aimed to: (I) establish a systematic compositional approach (0–100% R_m_:P_w_) for the development of optimal vermicompost formulations from mixed organic waste streams; (II) determine optimal ratios of P_w_:R_m_ that enhance the productivity of root, fruit, and leafy vegetables under sustainable production systems; (III) assess the combined effects of compost mixtures on soil biological, chemical, and physical properties in short- and long-term applications; (IV) integrate compost quality with soil health, crop productivity, and sustainability indicators to develop a comprehensive evaluation system; and (V) promote circular economy strategies by optimizing vermicomposting of mixed organic waste streams, thereby enhancing resource efficiency and waste recycling.

## Materials and methods

### Study area

The study was carried out over two consecutive seasons, for the years 2023–2024 and 2024–2025. The experiments for the study were carried out at the Agricultural Engineering Department, Faculty of Agriculture, Cairo University, Giza, Egypt, situated at a latitude of 30°17’ N and longitude of 31°12’ E and a mean altitude of 30 m above sea level.

### Reactor design

**Fig. 1:**
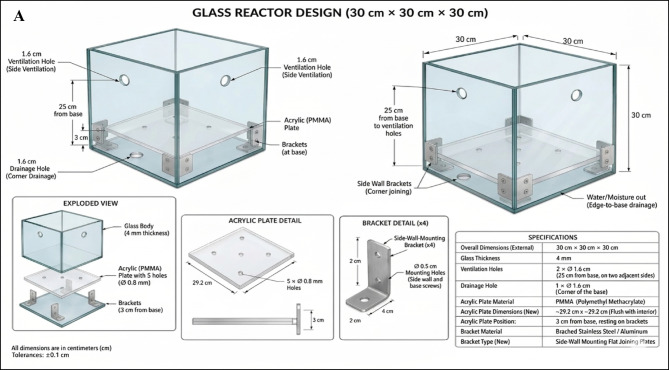

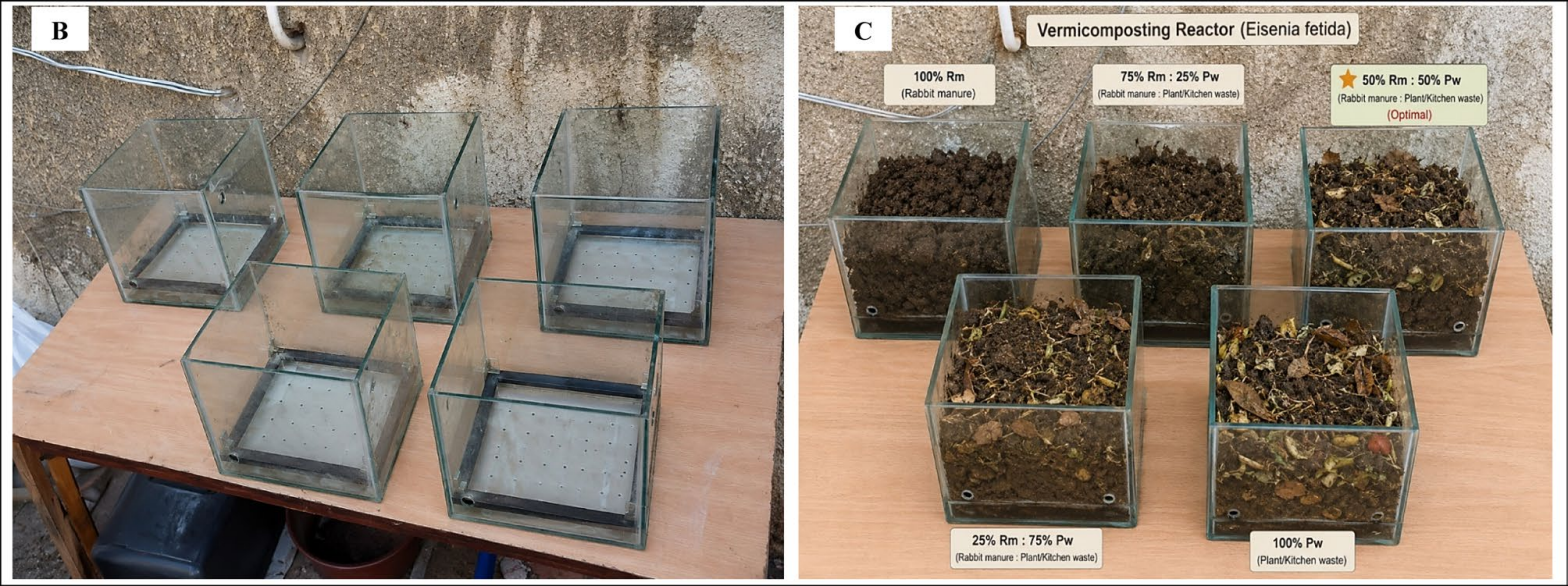
Experimental vermicomposting reactor system and feedstock treatments used in the study. (A) Schematic design and specifications of the glass vermicomposting reactor; (B) Photograph of the fabricated reactors prior to loading; (C) Reactors loaded with rabbit manure (R_m_) and plant/kitchen waste (P_w_) mixtures inoculated with Eisenia fetida under the five experimental treatments.

Glass reactors were designed as cubic boxes of equal dimensions (30 × 30 × 30 cm) with 4 mm of glass thickness (Fig. 1). Each box had three holes, 1.6 cm in diameter, and in their strategic locations to make them effective in their functions. There was a hole that was assigned in one corner of the reactor base to drain excess moisture. Conversely, the other two holes were positioned on two sides adjacent to each other at a height of 25 cm above the base to provide the required ventilation to the biological processes in the reactor. The plate (3 cm away from the reactor base) was attached using brackets to the inside walls of the reactor. To ensure a high humidity level to remove any extra water, the plate had five holes with a diameter of 0.8 mm.

### Vermicomposting production

The study was conducted using two types of organic wastes; fresh rabbit manure (R_m_) collected from a local rabbit farm was utilized as the source of animal-based organic matter. The plant materials were a heterogeneous mixture of agricultural and domestic residues (P_w_), which included wheat straw, fallen tree leaves, grass clippings, and household organic waste, the both wastes were selected to optimize C: N ratio and moisture balance. The rabbit and organic waste materials were manually shredded into pieces of lengths 2–5 cm to increase the surface area and allow microbial decomposition. The wastes were partially dried before application to give an optimum moisture content of between 55 and 60%.

The organic waste materials were placed in five composting reactors in predetermined ratios to obtain the desired level of compost production. Each reactor was assigned a fixed weight capacity of 3 kg, regardless of the 5 organic waste treatment types as follows: (1) Control, 100% R_m_ (3 kg), (2) 75% R_m_ (2.25 kg): 25% P_w_ (0.75 kg), (3) 50% R_m_ (1.5 kg): 50% P_w_ (1.5 kg), (4) 25% R_m_ (0.75 kg): 75%, P_w_ (2.25 kg), and (5) 100% P_w_ (3 kg) to evaluate how increasing proportions of kitchen waste influence substrate quality, nutrient dynamics, and vermicompost maturity while maintaining conditions suitable for earthworm activity. Each treatment was replicated three times, making a total of 15 reactors. All mixtures of organic waste went through a 15 days pre-composting period before earthworm inoculation. Pre-composting period was used to reduce heat and volatile compounds before worm introduction. This step helped to initiate initial thermophilic decomposition, minimized the accumulation of toxic gases (ex: ammonia), and stabilized the substrate, thus reducing phytotoxic effects and creating optimal conditions to support earthworm activity. After pre-composting, mature clitellate of the red wiggler, *Eisenia fetida* were added to each reactor.

*Eisenia fetida* was selected for this study due to its adaptability to warm, semi-arid climates in Giza, Egypt, its ease of reproduction, and its efficacy in processing manure-based and lignocellulosic organic waste feedstocks appropriate for the rabbit manure and plant/kitchen waste utilized in this research. It is the most accessible and one of the most extensively farmed epigeic earthworm species among local suppliers in Egypt, hence guaranteeing dependable sourcing for the experiment.

The earthworms were sourced to a local vermiculture supplier that was certified in Cairo, Faculty of Agriculture. A 50 adult earthworms’ inoculation was added to each reactor, which is equivalent to around 25 g of fresh biomass to provide a standardized stocking density commonly used in laboratory vermicomposting studies, ensuring sufficient decomposition activity without excessive competition. The earthworms were acclimatized before full inoculation with a small portion of their respective pre-composted substrate to reduce the environmental stress and improve the adaptation to the reactor conditions. The process took a time of 75 to 120 days depending on the composite of the waste mixture because previous studies have shown that this duration is generally sufficient for organic matter stabilization and vermicompost maturation under similar conditions. In the first active stage, there was a turning of the reactor contents twice a week, followed by once-a-week turning of the reactor contents during the maturation phase.

This was done through a rotary mechanical system that was fitted in each reactor. The moisture level was kept at 50–60% by means of irrigation as necessary because *Eisenia fetida* exhibits optimal growth, feeding, and cocoon production within this moisture range, with an automated spraying system built into the reactor lid within the optimal range for earthworm survival and microbial activity. To measure the rate of composting, temperature was measured daily at three vertical levels in each reactor (5 cm, 10 cm, and 20 cm) to maintain a homogenous heat distribution.

### Vermicomposting effectiveness on plant growth

To assess the effectiveness of the resulting compost on vegetable production and quality, three vegetable species were grown: tomato (*Solanum lycopersicum L. Cv. Moneymaker*), lettuce (*Lactuca sativa L. Cv. Grand Rapids)*, and carrot (*Daucus carota L. Cv. Nantes*).

The experiments in the greenhouse were carried out at the Faculty of Agriculture, Agricultural Engineering Department, Cairo University with a temperature range of 22–28 °C, relative humidity of 60–70%, and a photoperiod of 14 h. The substrate was sandy loam soil (68% sand, 18% silt, 14% clay) collected on the upper 20 cm of the experimental field at the faculty with an initial pH of 7.6, organic matter content of 1.2%, and available concentrations of the nutrients: 42 mg/kg nitrogen, 8 mg kg^− 1^ phosphorus, and 185 mg kg^− 1^ potassium. The mixture was placed in 10 L plastic pots (one plant per pot), which were put into the soil at a rate of 20% v/v (about 30 t ha^− 1^) based on equivalent agronomic application rates reported in previous vegetable production studies.

The treatments were planned in six repetitions within a completely randomized design (CRD). The plants were irrigated to achieve field capacity, but no additional chemical fertilizers were used. The tomato and lettuce experiments took 90 and 60 days after transplantation, respectively, and the carrot experiments required 100 days. The first measure of productivity was yield (kg ha^− 1^) measured on all crops. Tomato analysis was done by measuring the height of the plant (cm) and the quality index of the fruit to determine vegetative growth and marketable characteristics. Lettuce was evaluated based on the diameter of the plant (cm) and the nutrient content in the desiccated leaf tissue (mg/g), which was determined by chemical.

analysis of the dried leaf tissue. The morphological uniformity and the development of the root below the ground were measured by carrot root length (cm) and root diameter (cm).

### Environmental conditions

The experiment was conducted in a well-controlled greenhouse, and the local climate was described as dry, with a cold winter and a hot summer. The average environmental condition variables were recorded daily during both cultivation seasons (between October and February): air temperature in and out of the greenhouse, air relative humidity in and out, and sun radiation between October and February (Table 1).

**Table 1:** Monthly environmental condition variables in the greenhouse as an average for the two cultivated seasons.

Climate parameter	Month		
	December	January	February
T_in_ (^o^C)	28.32	27.78	28.52
T_out_ (^o^C)	22.05	19.90	19.42
RH_in_ (%)	65.43	63.78	64.62
RH_out_ (%)	62.47	58.36	56.17
Solar radiation (W m^− 2^)	72.58	79.36	97.17

### Irrigation water requirements

It is usually recommended that reference evapotranspiration (ETo) be computed utilizing the Penman-Monteith equation (Eq. 1), predicated on daily environmental condition metrics measured in greenhouse conditions. Equation (1) incorporates aerodynamic and thermodynamic characteristics, making it physically based and comprehensive. Having a model that can effectively represent how agricultural water requirements change in response to a changing climate is essential for hydrological modeling, water resource management, and precision irrigation scheduling in this study. Allen^25^ has effectively utilized the ET_o_. The weather data were input into the ET_o_ calculator software.

1 $$\:{ET}_{O}=\frac{0.408\varDelta\:\:\left({R}_{n}-G\right)+\gamma\:\left(\frac{900}{T+273}\right){U}_{2}({e}_{s}-{e}_{a})}{\varDelta\:+\gamma\:(1+0.34{U}_{2})}\:\:\:\:\:$$

where ET_o_: The reference evapotranspiration (mm day^− 1^), R_n_ : Net radiation at the crop surface (MJ m^-2^ day^− 1^), G : Soil heat flux density (MJ m^-2^ day^− 1^), T : The mean daily air temperature at 2 m height (°C), U_2_ : Wind speed at 2 m height (ms^− 1^). e_s_ and e_a_ are Saturation and Actual vapor pressure (kPa), respectively. ∆: Slope of the vapor pressure curve (kPa ºC^-1^). γ : Psychrometric constant (kPa ºC^-1^). The water requirements and watering schedule for the cucumber under a drip irrigation system were determined using the following equation (Eq. 2)^26^.

2 $$\:{IR}_{g}=\left(\frac{{ET}_{o}\times\:{K}_{C}\times\:{K}_{r}}{{E}_{i}}\right)-\left(R+LR\right)\:\:\:\:$$

where IR_g_ is the gross irrigation requirements (mm day^− 1^), ET_O_ is the reference evapotranspiration (mm day^− 1^), K_C_ is the crop coefficient, K_r_ represents the ground cover reduction factor, E_i_ is the irrigation efficiency (%), R represents the water received by plant from sources other than irrigation (mm), and LR is the amount of water required for the leaching of salts (mm). The values of K_r_ will be measured by Keller equation (Eq. 3)^27^.

3 $$\:{K}_{r}=GC+0.15\left(1-GC\right)\:\:\:\:\:\:\:\:\:\:$$

Where: GC is the ground cover (%) would be determined through dividing the shaded area per plant over the whole plant area.

### System installation and experimental treatments

**Fig. 2:**
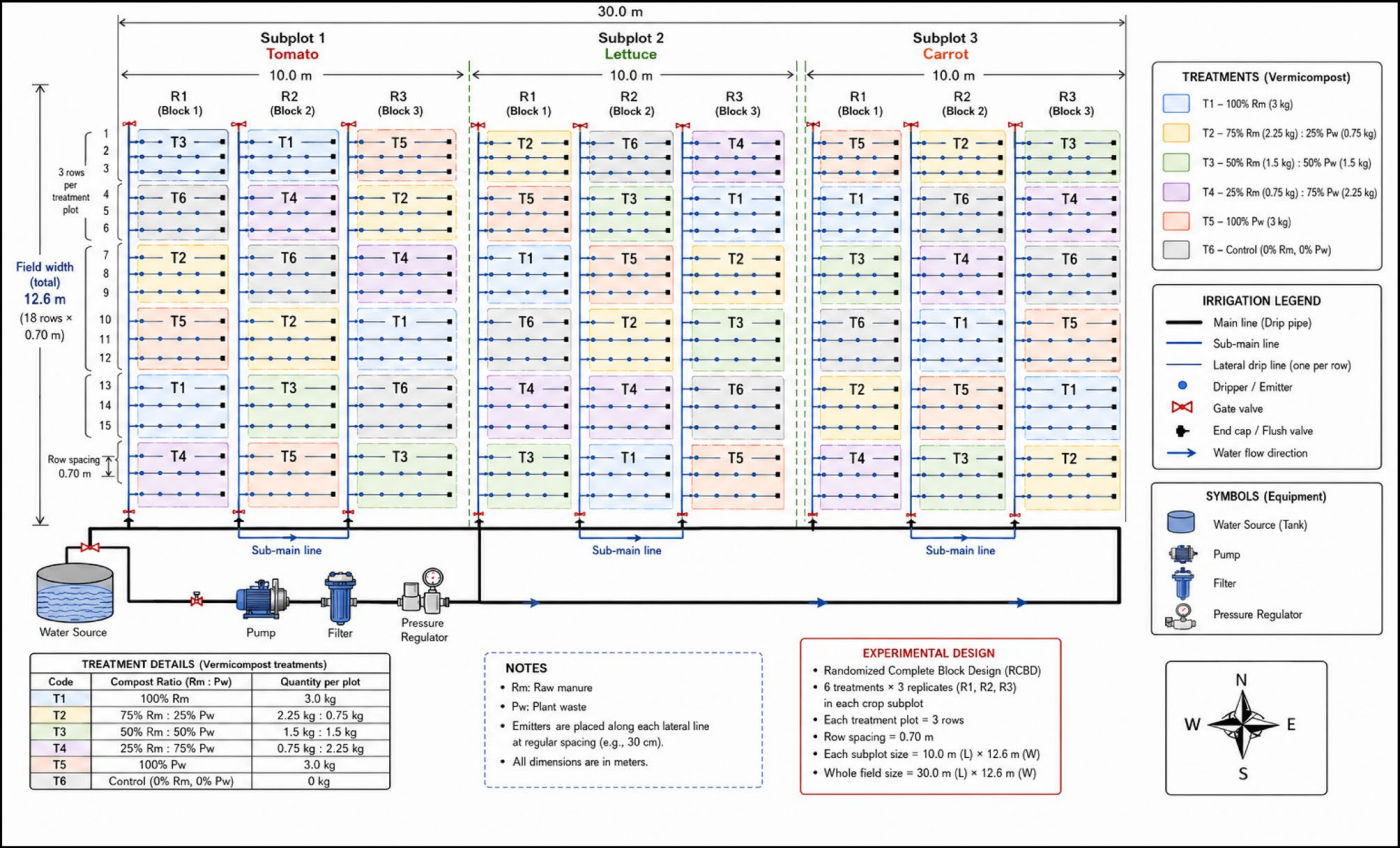
Experimental set-up layout diagram of the drip irrigation system and treatments.

The greenhouse experiment was conducted using 5 compost treatments, these treatments were applied on three vegetable crops: tomato, lettuce, and carrots. Using a drip irrigation system with a 10.5 m wide × 30 m length field plot was chosen for the experimental investigations. The field plot was split up into 3 subplots for each vegetable crop, with an area of 10.5 m wide × 10 m long subplots; each subplot consisted of 15 rows spaced 70 cm apart, and each sub-subplot consisted of 3 rows spaced 70 cm apart, indicating a single treatment with three replicates. The experiment was set up using a surface trickle irrigation system with a complete randomized block design. Three replicates of each treatment condition (R1, R2, and R3) were tested (Fig. 2).

### Measurements

#### Composting process indicators

The time required to reach peak temperature (°C) was recorded with a portable thermometer (Mextech Digital thermometer, Mextech, India) in days from the initiation of composting. The duration of the thermophilic phase was calculated as the number of days during which the temperature remained above 45 °C. Total composting time was defined as the number of days until stabilization of temperature, pH, and electrical conductivity. The percentages of mass and volume reduction were determined on the basis of the initial and final weight and volume of the compost.

#### Compost productivity and physicochemical properties

Productivity for each treatment was quantified using a digital balance with a precision of two decimal places. The following equation (Eq. 4) was applied to calculate compost productivity:

4 $$\:Productivity\:\left(\%\right)=\frac{\mathrm{M}\mathrm{a}\mathrm{s}\mathrm{s}\:\mathrm{o}\mathrm{f}\:\mathrm{p}\mathrm{r}\mathrm{o}\mathrm{d}\mathrm{u}\mathrm{c}\mathrm{e}\mathrm{d}\:\mathrm{c}\mathrm{o}\mathrm{m}\mathrm{p}\mathrm{o}\mathrm{s}\mathrm{t}\:}{\mathrm{M}\mathrm{a}\mathrm{s}\mathrm{s}\:\mathrm{o}\mathrm{f}\:\mathrm{f}\mathrm{r}\mathrm{e}\mathrm{s}\mathrm{h}\:\mathrm{w}\mathrm{a}\mathrm{s}\mathrm{t}\mathrm{e}}\times\:100\:\:\:\:\:\:\:\:\:\:$$

A PH meter (AD1000 Professional pH-ORP-TEMP Bench Meter with RS232/USB interface and GLP compliance) was used to measure the pH value in a 1:5 aqueous suspensions (compost: water). The chemical analysis was carried out to determine the total concentrations of macronutrients in the compost. Total nitrogen (TN) was determined by the Kjeldahl method, while total phosphorus (TP), potassium (TK), calcium (Ca), magnesium (Mg), and sulfur (S) were quantified using standard total digestion procedures. The values reported in this study therefore represent total nutrient concentrations on a dry-weight basis rather than water-extractable nutrient concentrations. These were measured on an RMX Raw Meal Analyzer that is capable of measuring elements in organic and inorganic materials with high precision. Electrical conductivity (EC) was measured in a 1:5 aqueous extract (compost: water) using an EC meter. The content of organic matter (percent) was estimated through loss-on-ignition by burning at 550 °C in 4 h according to Demir^28^.

Total carbon was assessed via the wet oxidation method (Walkley-Black procedure), whereas total nitrogen was measured using the Kjeldahl method. The carbon-to-nitrogen ratio (C: N) was subsequently determined. Demir^29^ measured bulk density using the usual cylinder method and estimated porosity based on the link between bulk density and particle density. The water retention capacity was evaluated using the osmotic pressure method.

The maturity of the compost was assessed by the germination index (GI), measured using seeds in accordance with a standardized bioassay. Ten seeds were positioned on filter paper saturated with 5 mL of aqueous compost extract (1:10, w/v) in Petri plates and incubated in darkness at 25 °C for 48 h. The germination rate and radicle elongation were measured and compared to a distilled water control. The germination index (GI) percentage was determined using Eq. 5.

5 $$\:GI\:\left(\%\right)=\left(\frac{\mathrm{G}\mathrm{e}\mathrm{r}\mathrm{m}\mathrm{i}\mathrm{n}\mathrm{a}\mathrm{t}\mathrm{i}\mathrm{o}\mathrm{n}\:\mathrm{p}\mathrm{e}\mathrm{r}\mathrm{c}\mathrm{e}\mathrm{n}\mathrm{t}\:\mathrm{i}\mathrm{n}\:\mathrm{c}\mathrm{o}\mathrm{m}\mathrm{p}\mathrm{o}\mathrm{s}\mathrm{t}\:\mathrm{e}\mathrm{x}\mathrm{t}\mathrm{r}\mathrm{a}\mathrm{c}\mathrm{t}\:}{\mathrm{G}\mathrm{e}\mathrm{r}\mathrm{m}\mathrm{i}\mathrm{n}\mathrm{a}\mathrm{t}\mathrm{i}\mathrm{o}\mathrm{n}\:\mathrm{p}\mathrm{e}\mathrm{r}\mathrm{c}\mathrm{e}\mathrm{n}\mathrm{t}\:\mathrm{i}\mathrm{n}\:\mathrm{c}\mathrm{o}\mathrm{n}\mathrm{t}\mathrm{r}\mathrm{o}\mathrm{l}}\right)\times\:\left(\frac{\mathrm{R}\mathrm{a}\mathrm{d}\mathrm{i}\mathrm{c}\mathrm{l}\mathrm{e}\:\mathrm{l}\mathrm{e}\mathrm{n}\mathrm{g}\mathrm{t}\mathrm{h}\:\mathrm{i}\mathrm{n}\:\mathrm{c}\mathrm{o}\mathrm{m}\mathrm{p}\mathrm{o}\mathrm{s}\mathrm{t}\:\mathrm{e}\mathrm{x}\mathrm{t}\mathrm{r}\mathrm{a}\mathrm{c}\mathrm{t}\:}{\mathrm{R}\mathrm{a}\mathrm{d}\mathrm{i}\mathrm{c}\mathrm{l}\mathrm{e}\:\mathrm{l}\mathrm{e}\mathrm{n}\mathrm{g}\mathrm{t}\mathrm{h}\:\mathrm{i}\mathrm{n}\:\mathrm{c}\mathrm{o}\mathrm{n}\mathrm{t}\mathrm{r}\mathrm{o}\mathrm{l}}\right)\times\:100$$

A GI value ≥ 80% was taken as the threshold for mature, non-phytotoxic compost. The ammonium-to-nitrate nitrogen ratio (NH₄⁺-N: NO₃⁻-N) was assessed as an additional stability measure. Ammonium nitrogen (NH₄⁺-N) was extracted using 2 MKCl and quantified calorimetrically by the indophenol-blue method, while nitrate nitrogen (NO₃⁻-N) was assessed through the cadmium reduction method. A ratio under 0.5 was employed as a benchmark for compost stability.

#### Plant growth parameters and total yield

40 days after transplantation occurred, the impact of different treatments on lettuce plant growth was assessed by measuring leaf area (LA) using a Biovis LA meter, counting the number of leaves, measuring plant height with a graduated meter, and determining plant fresh weight with a digital balance. The plants from each condition were individually collected at 60, 90, and 100 days post-transplanting for lettuce, tomato, and carrots, respectively, and weighed to ascertain total yield. The whole yield of vegetables was quantified in tons per acre.

#### SPAD index

The fifth leaf from four randomly selected plants per treatment was assessed for SPAD (chlorophyll content) using a portable chlorophyll meter (SPAD-520).

#### Determination of leaf nutrient and nitrate content

Freshly washed leaves were weighed and thereafter placed in a forced-air oven until a consistent dry weight was attained. Samples of 500 mg from each treatment were desiccated and ground into a fine powder. Sulfuric and perchloric acids (3:1) were employed to digest the crushed materials prior to chemical analysis. The percentages of nitrogen, phosphorus, potassium, and calcium in dry plant samples were determined according to the method described by Paez, Barrett^30^.

Macronutrients (N, P, K, Ca, Mg, S) were extracted via wet digestion and measured using suitable analytical methods. Total nitrogen was quantified using the Kjeldahl method, whilst phosphorus was assessed by the ammonium molybdate colorimetric method employing a spectrophotometer. The amounts of K, Ca, and Mg were evaluated via atomic absorption spectroscopy (AAS). The content of S was determined using the turbidimetric barium sulfate technique. The micronutrients (Fe, Mn, Zn, Cu, and B) were extracted using diethylene triaminepentaacetic acid (DTPA) and evaluated via inductively coupled plasma optical emission spectrometry (ICP-OES).

#### Biological properties

Bacteria, fungi, and actinomycetes populations were quantified on selective media using the serial dilution and plate count method, which was then examined under the microscope. Enzymatic activity was determined by measuring the activity levels of dehydrogenase, phosphatase, and protease enzymes, which were then combined to calculate the overall enzyme activity index. The effectiveness of pathogen suppression was tested by measuring the reduction in Escherichia coli and Salmonella populations using standard microbial enumeration techniques.

#### Physicochemical properties of soil after composting application

The pH, organic matter of the soil, the bulk density, and water-holding capacity were examined as described by Demir^29^, and the cation exchange capacity (CEC) was measured using the ammonium acetate exchange method^31^. Water retention capacity was measured between 0.33 bar (field capacity) and 15 bar (wilting point). Wet sieving was used to assess aggregate stability, while double-ring infiltrometers were used to determine infiltration rate.

#### Soil biological characteristics after composting application

The carbon content of microbial biomass was measured using the chloroform fumigation method. Dehydrogenase activity was measured. Earthworm populations were estimated by manual sorting inside a defined soil volume. To evaluate basal respiration, CO₂ retention was assessed. The fungal-to-bacterial ratio was determined using phospholipid fatty acid (PLFA) analysis.

#### Irrigation water productivity (IWP)

Water irrigation water productivity use efficiency (IWP) values were calculated according to the equations of Howell, Cuenca^32^(Eq. 6):

6 $$\:IWP=\frac{Y}{I}$$

Where: IWP is the irrigation water productivity (kg ha^− 1^), Y is the economic yield (kg ha^− 1^), and I is the irrigation water applied (m^3^ ha^− 1^).

### Statistical analyses

An experiment design with a completely random block and three replicates was used for each treatment. After integrating the data from the two growing seasons, homogeneity and normality tests were carried out. The pooled data from the two seasons were subjected to a Two-Way analysis of variance (ANOVA) using the IBM SPSS Statistical software program (version 25), followed by Duncan multiple range tests (*P* ≤ 0.05) to find significant differences across treatments. All of the data reported in the study are represented as mean values arising from interactions between different treatments, together with their respective standard errors (SE).

### Feasibility study

Annual total cost was calculated according to^33^ for the precision control system as follows (Eq. 7):

7 $${\text{Annual total cost}}\,=\,{\text{Total fixed costs}}\,+\,{\text{Total variable costs}}$$

Where the total fixed costs (US$ y^− 1^) include the costs of the control system, including the electrical board, Arduino, capacitance soil moisture sensors, solenoid valves, relay, adapter, memory card, and connecting wires and installation + additional infrastructure; total variable costs (US$ y^− 1^) equal total operational costs + energy costs + maintenance. The maintenance costs are taken as 2.2–7.4% of fixed costs (US$ y^− 1^) and years of working life expectancy (10 years).

## Results and discussions

### Vermicomposting process indicators and assessments

The composting process showed significant differences in dynamics based on the composition of the substrate, which is related to the effect of rabbit manure (R_m_) and plant/kitchen waste (P_w_) ratios on microbial activity and stability. The peak temperature was highest in the 100% Rm treatment (68 °C) at day 3, and lowest in the 100% P_w_ treatment (57 °C) at day 9. This suggests that rabbit manure, which is high in nitrogen and readily biodegradable organic matter, favors rapid microbial growth and heat production, while plant/kitchen waste alone degrades more slowly due to its lignocellulosic composition (Table 2).

The duration of thermophilic phase was highest in the 50% Rm: 50% P_w_ combination (25 days), indicating that the nutrient and moisture balance in this combination maintained optimal microbial growth for a longer time. Conversely, the thermophilic period was shortest (18 days) in 100% Rm, probably due to the depletion of substrates following initial microbial activity. This suggests the role of mixed substrates in stabilizing the composting process by providing sustained nutrient release and supporting microbial succession (Table 2).

**Table 2:** Comparative thermophilic composting characteristics of rabbit manure and plant/kitchen waste blends under vermicomposting conditions.

Treatment	Max temperature (^O^C)	Time to reach max temperature (day)	Duration of thermophilic phase (day)	Total composting time (day)
100% R_m_	68 ± 0.06 e	3.0 ± 0.07 a	18.0 ± 0.04 a	75.0 ± 0.02 a
75% R_m_:25% P_w_	65.0 ± 0.04 d	4.0 ± 0.10 b	21.0 ± 0.04 c	82.0 ± 0.04 b
50% R_m_:50% P_w_	63.0 ± 0.01 c	5.0 ± 0.06 c	25.0 ± 0.07 e	90.0 ± 0.02 c
25% R_m_:75% P_w_	60.0 ± 0.03 b	7.0 ± 0.01 d	22.0 ± 0.15 d	105.0 ± 0.04 d
100% P_w_	57.0 ± 0.12 a	9.0 ± 0.07 e	19.0 ± 0.12 b	120.0 ± 0.21 e

Means followed by different letters indicate significant differences between the treatments (*n* = 5; Duncan test at 95%).

The overall duration of composting time gradually increased with increasing P_w_ content, from 75 days in 100% R_m_ to 120 days in 100% P_w_. The longer time in P_w_ -rich treatments is due to the more recalcitrant fibrous structure and lignin content, which is difficult to break down. In contrast, manure-dominated treatments had a shorter time to maturity, highlighting the importance of nitrogen-rich feedstocks in promoting humification and stabilization (Table 2).

Overall, the findings show that mixed treatments, especially 50% R_m_: 50% P_w_, enhance composting efficiency by extending the duration of thermophilic phase while reaching maturity in a timely manner. This combination of quick heat build-up and sustained microbial activity is essential for producing safe, stable compost for use in agricultural production. The results highlight the significance of feedstock in vermicomposting and confirm the potential of rabbit manure as an accelerator when mixed with plant/kitchen waste.

**Fig. 3:**
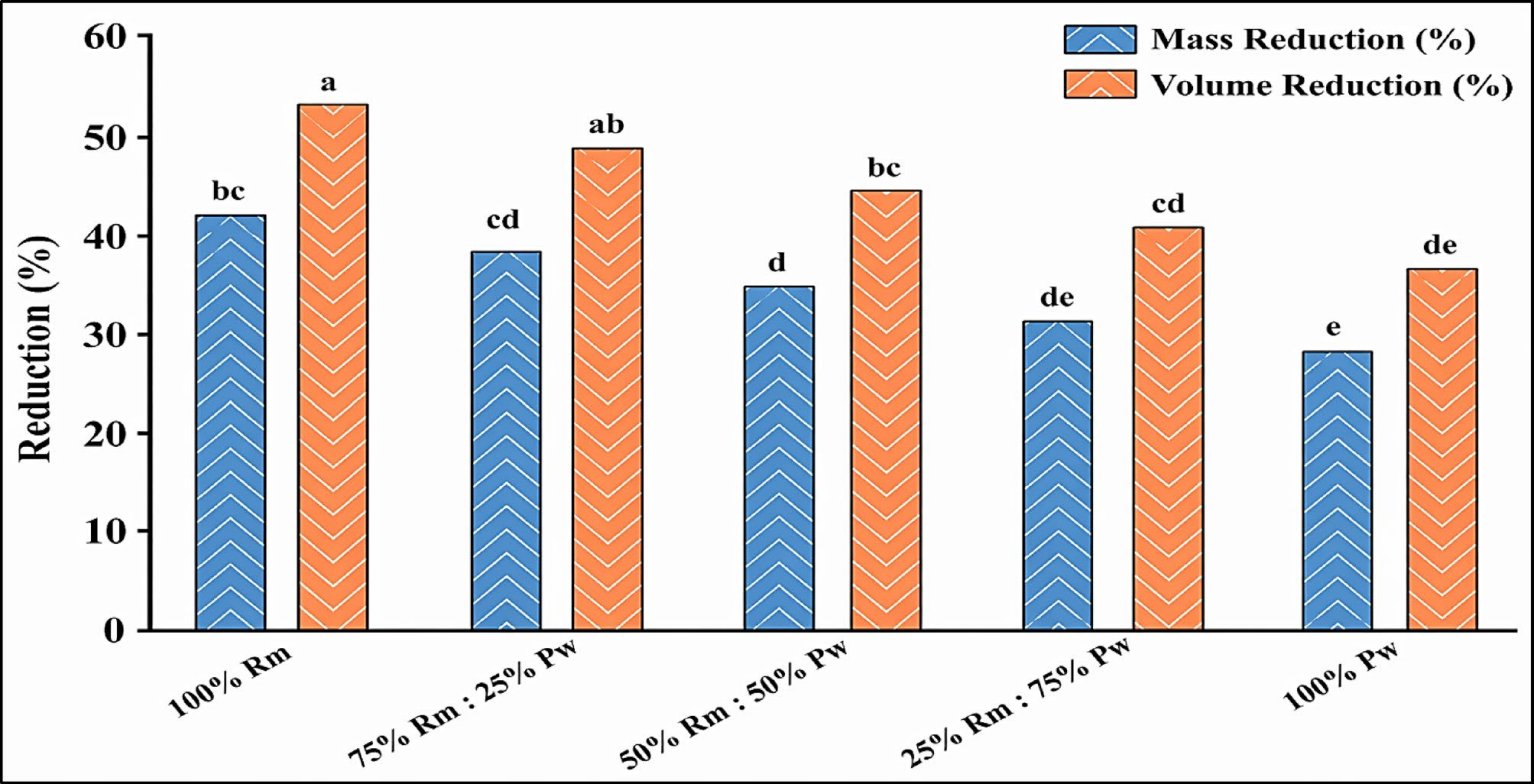
Effect of feedstock composition (R_m_: P_w_ ratios) on mass and volume reduction during vermicomposting with statistical grouping of treatments. Different letters indicate a significant difference between treatments (*n* = 5; Duncan test at 95%).

The findings (Fig. 3) show a gradual decrease in mass and volume reduction efficiencies with increasing P_w_ substitution for R_m_. Maximum reductions were seen in the 100% R_m_ treatment, while minimum reductions were seen in the 100% P_w_ treatment. The mixed 75:25 and 50:50 R_m_:P_w_ treatments exhibited intermediate values, suggesting that decomposition efficiency is highly dependent on the feedstock used. The mass reduction showed a gradual decrease from 100% R_m_ to 100% P_w_, with significant variations among treatments (indicated by different letters). This trend was also observed for volume reduction, although it was consistently greater than mass reduction for all treatments. This has been widely reported as a result of increased compaction and pore loss during vermicomposting, which results in more volume reduction than mass reduction. This is a common phenomenon, where physical fragmentation and microbial mineralization work together to stabilize the substrate (Fig. 3).

The better efficiency of R_m_-dominated treatments can be attributed to their more suitable biochemical properties, including optimal carbon-to-nitrogen ratios and biodegradability. Substrate properties are a key factor in determining the success of vermicomposting, by controlling microbial activity, earthworm feeding and organic matter decomposition^34^. Conversely, the lower efficiency in P_w_-dominated treatments could be linked to increased lignocellulosic material and recalcitrance, which can lead to slower degradation and lower mass loss.

The cluster analysis also reveals that treatments with higher R_m_ content (100% and 75%) belong to higher performance categories, while P_w_-dominated treatments (25% and 100%) are grouped into lower categories. This implies that a certain degree of P_w_ replacement with Rm can be achieved without severely compromising system performance, but a higher degree of replacement results in lower degradation efficiency (Fig. 3).

The finding results align with recent research published, for example, differences in waste combinations determine good performance of vermicomposting, where degradation and nutrient transformation are directly affected by the substrate used^35^. Further, the significance of earthworms in speeding up the degradation of organic matter via fragmentation, ingestion, and stimulation of microorganisms is well documented, leading to improved stabilization and reduction efficiencies^36^. The trends also agree with the general evidence that vermicomposting performance is lower when feedstocks have higher proportions of recalcitrant organic matter, which slows down the mineralization process.

In summary, the findings confirm that substrate ratios play a crucial role in achieving efficient vermicomposting. Though combined substrates can ensure acceptable efficiency, the predominance of easily degradable feedstocks (such as R_m_) is essential to attain higher mass and volume reduction. The results have implications for waste management practices, highlighting the selection and mixing of substrates to improve the efficiency and quality of the compost product.

### Effect of feedstock composition on compost physicochemical properties

Feedstock composition (raw manure (R_m_) and plant waste (P_w_) had a significant effect (*p* ≤ 0.05) on compost physicochemical properties (Table 3). There were distinct trends in all parameters measured, suggesting that feedstock composition is an important factor in compost quality.

#### pH and electrical conductivity (EC)

Compost pH decreased significantly from 8.3 ± 0.01 in 100% R_m_ to 6.2 ± 0.02 in 100% P_w_ (Table 3). This change from alkaline to neutral pH is due to the higher production of organic acids from the breakdown of plant/kitchen residues, and lower ammonification compared with manure-dominated compost. This is consistent with recent composting studies, where the addition of plant/kitchen residues alleviated compost alkalinity and enhanced its suitability for field application^37,38^.

**Table 3:** Physicochemical properties of vermicomposting.

Parameter	100% R_m_	75% R_m_ : 25% P_w_	50% R_m_ : 50% P_w_	25% R_m_ : 75% P_w_	100% P_w_
pH	8.3 ± 0.01 e	7.9 ± 0.01 d	7.4 ± 0.01 c	6.8 ± 0.02 b	6.2 ± 0.02 a
EC (dS/m)	5.8 ± 0.02 e	4.9 ± 0.01 d	3.7 ± 0.02 c	2.6 ± 0.02 b	1.8 ± 0.02 a
Organic matter (%)	37.2 ± 0.11 a	42.5 ± 0.01 b	48.7 ± 0.01 c	53.4 ± 0.02 d	58.1 ± 0.11 e
Bulk density (g/cm^3^)	0.62 ± 0.02 e	0.56 ± 0.02 d	0.48 ± 0.01 c	0.41 ± 0.01 b	0.35 ± 0.01 a
Water holding capacity (%)	145 ± 0.11 a	162.0 ± 0.01 b	178 ± 0.10 c	195 ± 0.04 d	210 ± 0.12 e
Porosity (%)	52 ± 0.05 a	58.0 ± 0.12 b	63 ± 0.11 c	69 ± 0.01 d	74 ± 0.16 e
Total nitrogen (%)	2.78 ± 0.04 e	2.4 ± 0.02 d	1.9 ± 0.03 c	1.4 ± 0.02 b	1.1 ± 0.02 a
Total carbon (%)	33.5 ± 0.021 a	36.1 ± 0.16 b	39.8 ± 0.14 c	37.8 ± 0.17 b	35.1 ± 0.19 ab
C/N ratio	12 ± 0.3 a	15.0 ± 0.4 b	21.0 ± 0.2 c	27 ± 0.3 d	32.0 ± 0.5 e
Compost productivity (%)	41 ± 0.6 a	38.0 ± 0.4 b	33.0 ± 0.5 c	30.0 ± 0.3 d	28.0 ± 0.5 e

Means followed by different letters indicate significant differences between the treatments (*n* = 5; Duncan test at 95%).

Electrical conductivity (EC) followed a similar decreasing trend, ranging from 5.8 ± 0.02 dS m⁻¹ in 100% R_m_ to 1.8 ± 0.02 dS m⁻¹ in 100% P_w_. Elevated EC in manure-rich compost is due to soluble salts from mineralization, while plant/kitchen residues reduce salinity. EC values below 4 dS m⁻¹, such as in treatments with ≥ 50% P_w_, are safe for plant growth and indicate low salinity^39^.

#### Organic matter, bulk density, and porosity

Organic matter (OM) content increased from 37.2 ± 0.11% (100% R_m_) to 58.1 ± 0.11% (100% P_w_), due to the higher lignocellulosic content of plant waste. This promotes humification and carbon stabilization, which play a crucial role in enhancing soil fertility^40^.

Bulk density decreased from 0.62 ± 0.02 g cm^-^³ to 0.35 ± 0.01 g cm^-^³, while porosity increased from 52 ± 0.05% to 74 ± 0.16% with increasing P_w_ proportion (Table 3). This reflects better soil structure, with increased air permeability and decreased bulk density. These changes play essential roles in microbial growth and plant root development, especially in degraded soils^41^. The water holding capacity (WHC) also improved from 145 ± 0.11% to 210 ± 0.12%, showing the enhanced water retention capacity (WRC) of plant-based composts. This is mainly because of the higher organic matter and pore volume, which improve water adsorption and WHC^38^.

#### Total nitrogen, total carbon, and C/N ratio

Total nitrogen (TN) content dropped from 2.78 ± 0.04% in 100% R_m_ to 1.1 ± 0.02% in 100% P_w_, with manure having a higher nitrogen content. However, total carbon (TC) generally increased to a peak value in the 50% R_m_: 50% P_w_ treatment (39.8 ± 0.14%) and then decreased slightly in higher P_w_ treatments (Table 3).

The C/N ratio gradually rose from 12 ± 0.3 to 32 ± 0.5 with higher P_w_ levels. The lower C/N ratios (< 15) in manure-dominated treatments suggest fast mineralization and potential nitrogen loss via volatilization. On the other hand, higher C/N ratios (> 25) in plant-dominated treatments indicate slow decomposition and potential nitrogen immobilization (Table 3).

The 50% R_m_:50% P_w_ (21 ± 0.2) and 25% R_m_:75% P_w_ (27 ± 0.3) treatments have a C/N ratio in the optimal range (20–30) for mature compost, suggesting balanced microbial activity and effective organic matter transformation^37,39^.

#### Compost productivity

Compost productivity decreased significantly from 41 ± 0.6% in 100% R_m_ to 28 ± 0.5% in 100% P_w_ (Table 3). The increased yield of manure-based compost was due to the quick decomposition of easily degradable organic matter. Although P_w_-based compost was less productive, it had better physicochemical properties, suggesting that compost quality does not depend only on productivity but also on maturity and stability^38^.

#### Optimization of compost formulation

Among the tested treatments, the 50% R_m_: 50% P_w_ mixture demonstrated the most balanced characteristics, including pH (7.4 ± 0.01), EC (3.7 ± 0.02 dS m⁻¹), OM (48.7 ± 0.01%), and C/N ratio (21 ± 0.2) (Table 3). These levels suggest the compost is mature, well-balanced in nutrients, and has an ideal structure. The mixture of manure and plant waste promotes microbial activity through the availability of easily degradable nitrogen and stable carbon, facilitating decomposition and humification. Recent research stresses the significance of mixed feedstocks for enhancing compost stability, nutrient release, and effectiveness as a soil amendment by improving the quality of compost compared to single-source composts^41^.

#### Implications for sustainable soil management

The study shows that combining plant/kitchen waste and manure can enhance compost quality. Higher P_w_ ratio: Lower salinity and alkalinity, higher organic matter, better physical properties (porosity and WHC) and C/N ratio (Table 3). These changes are important for sustainable farming, which relies on preserving soil organic matter and minimizing environmental pressures. Composting of balanced organic waste mixtures has been reported to improve soil fertility, microbial community, and soil productivity^42^.

### Macronutrient dynamics as affected by feedstock composition

**Fig. 4:**
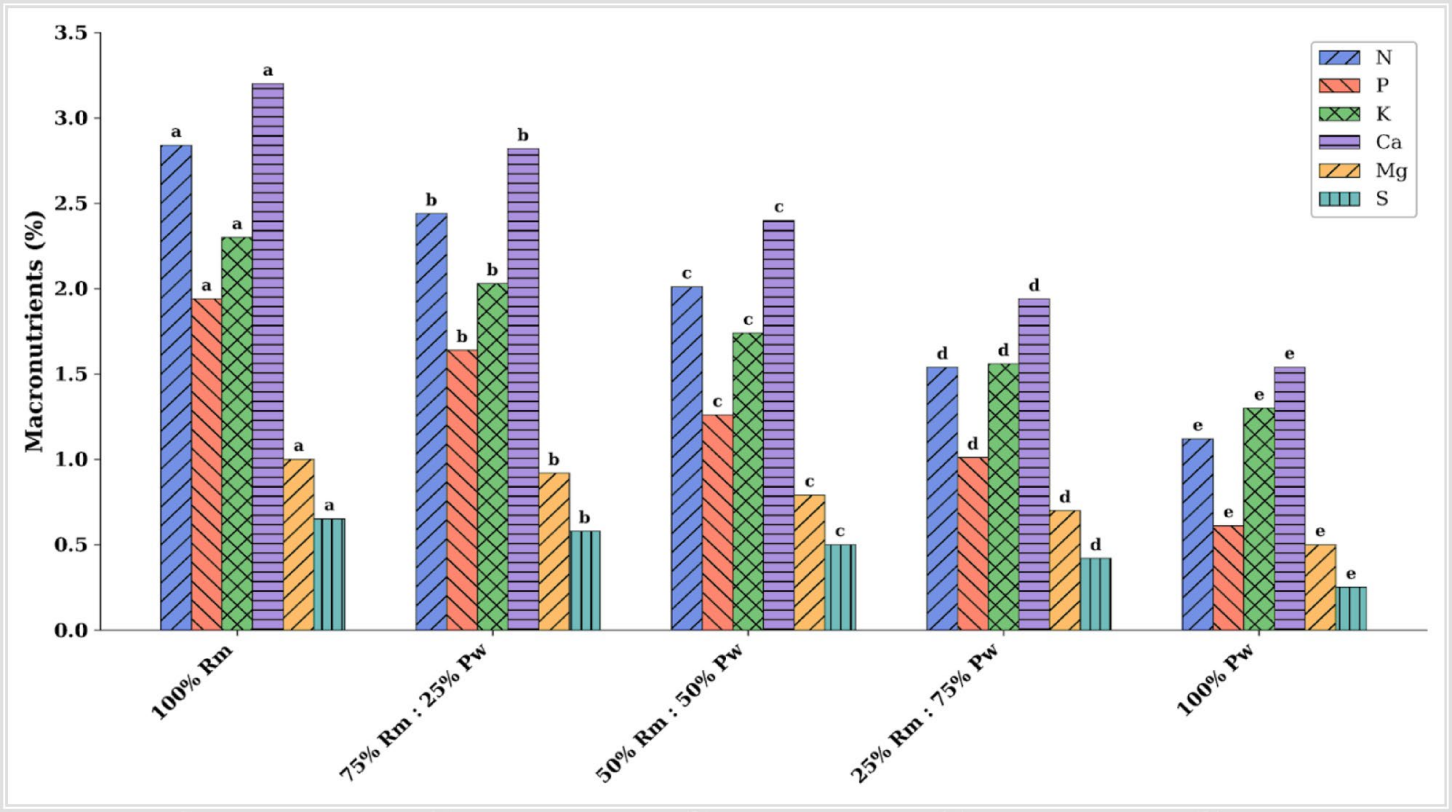
Comparative analysis of mineral macronutrient profiles (N, P, K, Ca, Mg, and S) in vermicompost under varying rabbit manure and plant/kitchen waste blends. Different letters indicate a significant difference between treatments (*n* = 5; Duncan test at 95%).

The content of macronutrients in vermicompost changed consistently with the ratio of plant/kitchen residues (P_w_) and rabbit manure (R_m_) (Fig. 4). In all cases, nutrient concentrations decreased as P_w_ increased.

Total nitrogen (N) content ranged from 2.8% in 100% R_m_ to 1.1% in 100% P_w_, a decline of 61%. Intermediate mixtures showed a gradual decline, with values of 2.4%, 2.0%, and 1.5% in 75% R_m_: 25% P_w_, 50% R_m_: 50% P_w_, and 25% R_m_: 75% P_w_, respectively. Nitrogen (N) concentration decreased from 100% R_m_ treatment (~ 2.8%) to 100% P_w_ treatment (~ 1.1%) (Fig. 4). This is attributed to higher initial N content and mineralization rate of rabbit manure than the lignocellulosic plant/kitchen residues. Manure-rich treatments increase microbial activity and N mineralization, leading to higher N content in vermicompost. This trend is due to slower mineralization and increased nitrogen immobilization in these C-rich substrates. Such a decline has also been reported in vermicomposting systems, where lignocellulosic substrates dilute total N and reduce degradation rates^12^.

Similarly, phosphorus (P) and potassium (K) concentrations showed a decreasing trend, with higher levels in treatments with higher Rm content. The higher P content in treatments containing manure is in line with increased phosphatase activity in earthworm casts, which facilitates P mineralization^43^. Phosphorus (P) concentrations decreased from 2.3% (100% R_m_) to 1.2% (100% P_w_), with intermediate values of 2.0%, 1.7%, and 1.4%. The increased P concentration in manure-based mixtures is explained by higher phosphatase activity and P mineralization during the vermicomposting process. Similarly, potassium (K) decreased from 1.9% to 0.6% for the same treatments. The gradual decrease in K content may be due to dilution and reduced mineral release from plant-based substrates. Similarly, K is highly dependent on the mineral component of the substrate and is generally higher in fertile organic wastes. The progressive reduction in P and K in P_w_-dominated treatments is likely due to dilution and low mineral release from plant/kitchen wastes (Fig. 4).

The trends for secondary nutrients (Ca, Mg and S) were similar. Calcium showed the highest concentration in all treatments, but declined with the addition of P_w_. This could be due to the release of Ca-rich calcareous matter by earthworms, and the concentration effect during organic matter decomposition, which is more significant in manure-based media^44^. Calcium (Ca) was the most abundant macronutrient in the treatments, ranging from 3.2% (100% R_m_) to 1.5% (100% P_w_). The mixed treatments (25% P_w_: 75% R_m_, 50% P_w_: 50% R_m_, and 75% P_w_: 25% R_m_) had concentrations of 2.8%, 2.4%, and 1.9%, respectively, showing high levels of Ca retention even at low P_w_ concentrations. The higher Ca levels in manure treatments may be linked to the substrate characteristics and concentration effects by earthworms during the decomposition of organic matter (Fig. 4).

The concentrations of magnesium (Mg) and Sulphur (S) were relatively lower but showed a similar decrease. Mg concentrations declined from 0.9% to 0.4% and S from 0.6% to 0.2% respectively along the gradient. These declines are in line with secondary nutrients being dependent on substrate quality and mineralization. The decrease in Mg and S content with Pw also reflects the impact of substrate mineral content and microbial transformation. Other studies have reported increased Ca, Mg and S concentrations in vermicompost compared to substrates, due to mass loss and nutrient transformation^45^.

#### Implications for substrate optimization

The treatment rankings (100% R_m_ > 75% R_m_: 25% P_w_ > 50% R_m_: 50% P_w_ > 25% R_m_: 75% P_w_ > 100% P_w_) for all macronutrients indicate the primary influence of manure on nutrient concentration. But while the 100% R_m_ treatment provided the highest nutrient levels, the blended substrates (especially 50–75% R_m_) retained high levels of nutrients while also potentially enhancing the physicochemical characteristics of the compost (air, porosity, stability of organic matter).

The 50% R_m_: 50% P_w_ treatment, for example, maintained ~ 71% of total N and ~ 75% of Ca concentrations compared to the 100% R_m_ treatment, indicating that mixing with plant/kitchen residues does not significantly reduce nutrient levels. Rather it may lead to a more balanced compost product, with an improved C/N ratio and microbial activity. On the other hand, the 100% P_w_ treatment consistently showed the lowest nutrient content, suggesting that it may not be suitable for applications requiring high nutrient content^12^.

Agronomically, the findings demonstrate that vermicompost produced from mixed manures (especially 50–75% R_m_) has a more desirable nutrient profile, containing moderate to high macronutrient concentrations, while also improving organic matter properties. Although using 100% manure has the highest nutrient content, it may cause problems like salinity or nutritional imbalance. On the other hand, using high P_w_ ratios enhances structural quality but also decreases nutrient concentrations. So, the optimal balance between R_m_ and P_w_ is crucial in achieving both fertility and sustainability targets in vermicompost.

In summary, the variability observed confirms that feedstock management controls the nutrient dynamics in vermicomposting, largely by affecting microbial activity, nutrient mineralization, and organic matter stability. These results are in line with recent research highlighting the importance of vermicompost as a slow-release, nutrient-rich fertilizer whose characteristics can be controlled by feedstock composition^12^.

### Micronutrient dynamics as affected by feedstock composition

Micronutrients (Fe, Mn, Zn, Cu, and B) concentration showed significant (*p* < 0.05) variations among the treatments (Table 4), highlighting the effects of feedstock composition on nutrient enrichment in vermicomposting. An increasing trend was observed for Fe, Mn, Zn, and Cu concentrations with increasing proportions of plant/kitchen residues (P_w_) while boron (B) showed the opposite.

Iron (Fe) showed the highest concentration among all, ranging from 3950 ± 0.22 mg kg⁻¹ in 100% R_m_ (a) to 2470 ± 0.05 mg kg⁻¹ in 100% P_w_ (e). The intermediate treatments had concentrations of 3580 ± 0.03 (b), 3210 ± 0.04 (c), and 2840 ± 0.04 mg kg⁻^1^ (d), showing a statistically significant decrease from the gradient. The higher Fe content in manure-based treatments is typical of increased mineralisation and concentration resulting from the breakdown of organic matter. Similar trends in Fe increase in vermicompost have been linked to the conversion of organic fractions to more stable forms during earthworm processing^46^.

Manganese (Mn) followed a comparable decreasing trend, ranging from 425 ± 0.04 mg kg⁻¹ (a) in 100% R_m_ to 215 ± 0.05 mg kg⁻¹ (e) in 100% P_w_. Zinc (Zn) and copper (Cu) exhibited parallel patterns, decreasing from 187 ± 0.14 to 60 ± 0.15 mg kg⁻¹ and 65 ± 0.02 to 21 ± 0.08 mg kg⁻¹, respectively. The decreasing trend in these is due to dilution and lower initial concentrations of micronutrients in plant/kitchen residues than manure. Additionally, vermicomposting leads to the release and redistribution of micronutrients like Zn and Cu due to the biotransformation by earthworms and microbial activities^47^.

In contrast, boron (B) increased significantly with increasing P_w_ proportion, ranging from 32 ± 0.05 mg kg⁻¹ (e) in 100% R_m_ to 44 ± 0.11 mg kg⁻¹ (a) in 100% P_w_. Intermediate treatments showed a steady increase (35 ± 0.07, 38 ± 0.07, and 41 ± 0.13 mg kg⁻¹). This inverse correlation shows that the plant/kitchen residues are a relatively good source of B, and its release increases during lignocellulosic matter decomposition. Increases in B concentrations during vermicomposting have been attributed to organic matter mineralization and the development of soluble micronutrient pools^48^.

#### Implications for nutrient enrichment and substrate optimization

The order of treatments for Fe, Mn, Zn and Cu 100% R_m_ > 75% R_m_ > 50% R_m_ > 25% R_m_ > 100% P_w_) corroborates that manure-based substrates are the main source of micronutrient enrichment (Table 4). This finding is in line with previous reports that nutrient concentrations of vermicompost are primarily determined by feedstock and increased by microbial and earthworm activity^49^.

Although the highest concentrations of micronutrients were found in 100% R_m_, the 50% R_m_: 50% P_w_ mix still contained significant levels of micronutrients (81% Fe, 75% Mn, 66% Zn and 66% Cu) compared to 100% R_m_. This suggests that replacing part of manure with plant/kitchen residues will not dramatically affect micronutrient content, but may enhance compost stability and structure (Table 4).

The rising B content with increasing P_w_ addition is a valuable supplement, implying that blends can result in a better-balanced micronutrient content. From an agricultural standpoint, this is desirable as micronutrients such as Fe, Zn, Mn, and Cu are necessary for a range of plant physiological processes such as enzymatic activation, chlorophyll formation and nutrient metabolism^50^.

Most of the micronutrients are enriched by the animal’s feed and metabolism, which is probably why the concentrations of most micronutrients are higher in manure-based fertilizers. The concentration of all the micronutrients except boron is higher in the plant materials used for compost than the animal feed and hence the manure. On the other hand, in agricultural use, the micronutrient composition of the manure-based fertilizers makes them particularly valuable in overcoming the micronutrient deficiencies, particularly in soils deficient in zinc or copper. These organic amendments supply micronutrients directly while also improving nutrient availability through changes in soil physicochemical and biological properties. During decomposition, organic matter releases Zn and Cu gradually and forms soluble organic complexes that reduce nutrient fixation and enhance micronutrient mobility in the rhizosphere. Additionally, increased microbial activity associated with organic amendments can promote nutrient mineralization and facilitate plant uptake. Zinc is essential for enzyme activation, protein synthesis, and plant growth regulation, whereas copper plays important roles in photosynthesis, respiration, and oxidative metabolism.

**Table 4:** Effect of feedstock composition on micronutrient content of vermicompost produced from rabbit manure (R_m_) and plant/kitchen residues (P_w_).

Treatment	Iron	Manganese	Zinc	Copper	Boron
100% R_m_	3950 ± 0.22 a	425 ± 0.04 a	187 ± 0.14 a	65 ± 0.02 a	32 ± 0.05 e
75% R_m_ : 25% P_w_	3580 ± 0.03 b	372 ± 0.04 b	156 ± 0.12 b	54 ± 0.02 b	35 ± 0.07 d
50% R_m_ : 50% P_w_	3210 ± 0.04 c	320 ± 0.13 c	124 ± 0.07 c	43 ± 0.09 c	38 ± 0.07 c
25% R_m_ : 75% P_w_	2840 ± 0.04 d	268 ± 0.03 d	92 ± 0.01 d	32 ± 0.05 d	41 ± 0.13 b
100% P_w_	2470 ± 0.05 e	215 ± 0.05 e	60 ± 0.15 e	21 ± 0.08 e	44 ± 0.11 a

Therefore, the application of manure-derived vermicompost may improve micronutrient availability and support vegetable productivity, particularly in micronutrient-deficient. However, if the copper and zinc are applied at high rates in the application, there could be a concern about the accumulation of these in the soil, particularly if the fertilizer is applied intermittently^51^. The 50/50 combination of R_m_ and P_w_ is a compromise between relatively high concentrations of all the micronutrients, and can be used safely in most agricultural applications without the risk of deficiency or toxicity. In conclusion, the findings show that the nature of the feedstock used is a key factor affecting the micronutrient dynamics during the vermicomposting process, with a combination of organic wastes (50–75% R_m_) being the best option in terms of both micronutrient enrichment and sustainable waste management.

### Biological properties as influenced by feedstock composition

**Fig. 5:**
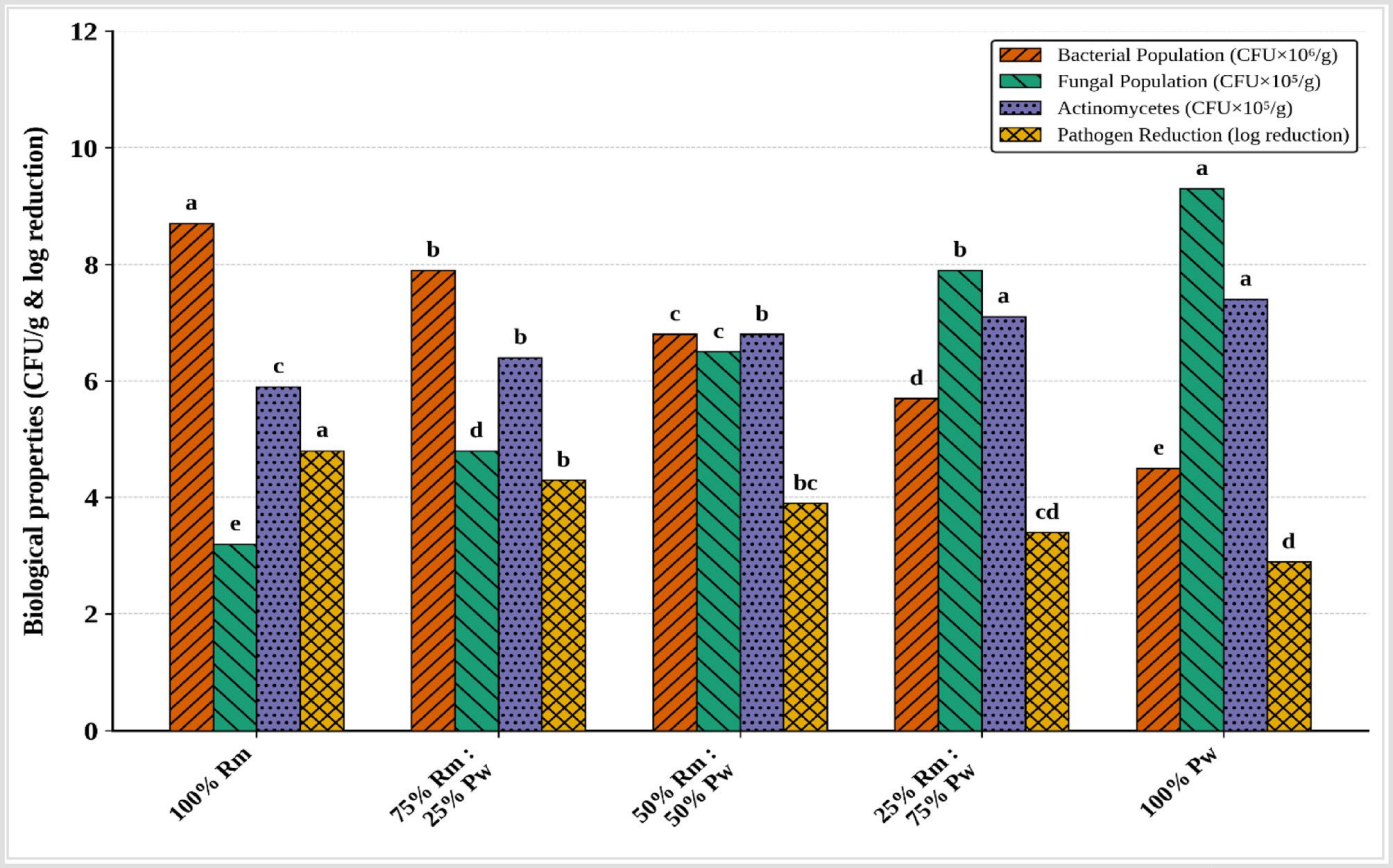
Microbial community dynamics and pathogen suppression in vermicompost derived from varying ratios of animal manure and plant waste. Different letters indicate a significant difference between treatments (*n* = 5; Duncan test at 95%).

The influence of rabbit manure (R_m_) and plant/kitchen waste (P_w_) proportions on the biological properties of vermicompost, such as bacterial, fungal, and actinomycetes numbers and pathogen reduction, is shown in Fig. 5. Various patterns were observed in the treatments, reflecting microbial changes during the vermicomposting process.

Bacterial counts (CFU × 10⁶ g⁻¹) gradually declined with the proportion of P_w_, from 8.7 in 100% Rm to 4.5 in 100% P_w_ (a 48% reduction). Intermediate treatments recorded 7.9, 6.8, and 5.7 in 75% R_m_: 25% P_w_, 50% R_m_: 50% P_w_, and 25% R_m_: 75% P_w_, respectively. The greater bacterial population in manure-rich media is in line with the presence of readily available organic matter that encourages bacterial growth. Bacteria are typically predominant in vermicomposting and are involved in the degradation of organic matter and mineralization of nutrients^52^.

In contrast, the abundance of fungi increased with increasing P_w_ content from 3.2 (100% R_m_) to 9.3 (100% P_w_). The intermediate values were 4.8, 6.5 and 7.9, respectively. This change is related to the preference of fungi for lignocellulosic plant/kitchen residues, since fungi are better adapted to decompose complex polymers like cellulose and lignin. Recent research indicated that vermicomposting can shift microbial populations, frequently resulting in an increase of fungal diversity in carbon-rich substrates and improved functional diversity in bacterial populations^53^.

The actinomycetes population showed a gradual increase, from 5.9 (100% R_m_) to 7.4 (100% P_w_), with values of 6.4, 6.8 and 7.1 in between. The progressive rise in actinomycetes with increasing P_w_ is probably due to their involvement in the decomposition of recalcitrant organic matter. They play a crucial role in organic matter stabilization and production of bioactive substances beneficial for soil health (Fig. 5).

The pathogen reduction (log reduction) was negatively correlated with P_w_ percentage, with values ranging from 4.8 in 100% R_m_ to 2.9 in 100% P_w_. The mixed treatments had 4.3, 3.9 and 3.4, suggesting a reduction in sanitization efficiency with increasing plant residue in the mixture. This greater pathogen reduction in manure-based treatments is due to increased microbial competition, enzyme activity, and earthworm gut processes. Vermicomposting has been well documented to decrease pathogenic microbes, such as Escherichia coli and Salmonella, through direct ingestion and indirect antagonism^54^. Recent studies also show vermicomposting successfully removes human pathogens, and changes microbial communities to produce safer compost^53^.

#### Implications for microbial balance and compost safety

These results suggest a trade-off between microbial populations and community structure. Manure-rich substrates supported high bacterial abundance and pathogen reduction, while P_w_-rich substrates supported fungal and actinomycetes dominance, which is indicative of a transition to slower decomposition processes. This shift is in line with the succession of microbial communities during vermicomposting, in which the quality of the substrate controls the structure and function of the microbial community (Fig. 5).

While the 100% R_m_ treatment showed the highest bacterial activity (8.7) and pathogen reduction (4.8 log units), the 50% R_m_: 50% P_w_ treatment offered a balanced microbial community, with high bacterial (6.8), fungal (6.5) and actinomycetes (6.8) populations and satisfactory pathogen reduction (3.9). This is preferred for producing stable compost both in terms of microbial activity and safety.

On the other hand, the 100% P_w_ treatment, while stimulating the highest fungal (9.3) and actinomycetes (7.4) populations, exhibited the lowest pathogen reduction (2.9), indicating poorer sanitization. This suggests the need to balance nutrient-rich and carbon-rich substrates to achieve optimal microbial diversity and safety (Fig. 5).

Overall, the results demonstrate that substrate quality plays a key role in regulating microbial activity and pathogen reduction during vermicomposting, with mixed feeds (especially 50–75% R_m_) providing the best compromise between biological activity, decomposition and compost safety.

### Effect of vermicomposting on vegetables yield and water productivity

The ANOVA showed that yield, water applied and WP were significantly influenced (*p* ≤ 0.05) by vermicompost treatments for all the vegetables (tomato, lettuce and carrot) (Table 5). A further comparison of the means, using the least significant difference (LSD) test at *p* ≤ 0.05, showed significant differences among treatments, as shown by different letters.

#### Tomato

Vermicompost treatments had a significant effect on tomato yield and WP. The highest yield (102,000 kg ha⁻¹) and maximum WP (16.19 kg m⁻³) was obtained under the 50% R_m_: 50% P_w_ treatment, which was significantly (*p* ≤ 0.05) better than other treatments. Conversely, the lowest yield (51,000 kg ha⁻¹) and WP (7.36 kg m⁻³) were observed under the control treatment, showing a 50% yield reduction compared to the best treatment. Water application varied across treatments, with the maximum water applied (7245 m³ ha⁻¹) under 100% R_m_, and the minimum (5606 m³ ha⁻¹) under 100% P_w_ treatment. But increased water use did not lead to increased productivity, as indicated by the reduced WP under 100% R_m_.

The better performance of 50% R_m_: 50% P_w_ can be linked to the combined effect of balanced nutrient supply and improved soil physical properties leading to better water holding capacity and nutrient availability. This observation aligns with recent research that shows combined vermicompost applications enhance tomato productivity and WP under drip irrigation^55,56^.

#### Lettuce

Similar to tomato, vermicompost treatments significantly influenced the yield and WP of lettuce (*p* ≤ 0.05). The highest yield (45,000 kg ha⁻¹) and WP (21.72 kg m⁻³) were recorded with 50% R_m_: 50% P_w_, followed by 25% R_m_: 75% P_w_, which did not differ significantly in WP (shared statistical grouping). In contrast, the lowest yield (24,000 kg ha⁻¹) and WP (10.53 kg m⁻³) were found in the control treatment.

Water use varied from 2383 m³ ha⁻¹ (100% R_m_) to 1844 m³ ha⁻¹ (100% P_w_) with significant differences. The treatments with higher levels of plant waste produced higher WP, despite using less water, suggesting improved water-use efficiency.

The better performance of the mixed vermicompost treatments is probably related to increased microbial activity, mineralization and water-holding capacity, which are essential for the growth of leafy vegetables. This is consistent with earlier studies that showed that vermicompost applications greatly enhance lettuce yield and resource-use efficiency^14,57^.

#### Carrot

The response of carrot was different from tomato and lettuce. The maximum yield (57,000 kg ha⁻¹) and WP (14.62 kg m⁻³) were recorded under 25% R_m_: 75% P_w_, and were significantly greater (*p* ≤ 0.05) than the other treatments. Once again, the lowest yield (35,000 kg ha⁻¹) and WP (7.66 kg m⁻³) were recorded in the control treatment.

**Table 5:** Effect of different vermicompost media on yield and water productivity of vegetable crops.

Treatment	Tomato			Lettuce				Carrot			
	Yield (Kg ha^− 1^)	Water applied (m^3^ ha^− 1^)	Water productivity (Kg m^− 3^)	Yield (Kg ha^− 1^)		Water applied (m^3^ ha^− 1^)	Water productivity (Kg m^− 3^)	Yield (Kg ha^− 1^)		Water applied (m^3^ ha^− 1^)	Water productivity (Kg m^− 3^)
100% R_m_	84,000 ± 0.50d	7245 ± 0.91a	11.59 ± 0.05e	36,000 ± 1.05e		2383 ± 0.58a	15.11 ± 0.11d	42,000 ± 1.73e		4774 ± 0.22a	8.79 ± 0.09e
75% R_m_ + 25% P_w_	96,000 ± 1.91b	6804 ± 0.52c	14.11 ± 0.12c	42,000 ± 0.93b		2237 ± 0.14c	18.78 ± 0.72c	51,000 ± 0.14d		4483 ± 0.56c	11.38 ± 0.05d
50% R_m_ + 50% P_w_	102,000 ± 1.59a	6300 ± 0.88d	16.19 ± 0.21a	45,000 ± 0.31a		2072 ± 59d	21.72 ± 0.12a	54,000 ± 0.13b		4151 ± 0.97d	13.01 ± 0.7c
25% R_m_ + 75% P_w_	89,000 ± 0.53c	5921 ± 0.66e	15.03 ± 0.17b	41,000 ± 0.74c		1949 ± 0.92e	21.04 ± 0.25a	57,000 ± 0.42a		3900 ± 0.12e	14.62 ± 0.55a
100% P_w_	76,000 ± 0.19e	5606 ± 0.17f	13.56 ± 0.32d	37,000 ± 0.11d		1844 ± 0.16f	20.07 ± 0.55b	52,000 ± 0.07c		3695 ± 0.21f	14.07 ± 0.23b
Control	51,000 ± 0.96f	6930 ± 0.45b	7.36 ± 0.17f	24,000 ± 0.42f		2279 ± 0.61b	10.53 ± 0.63e	35,000 ± 0.93f		4567 ± 0.47b	7.66 ± 0.09f

Means followed by different letters indicate significant differences between the treatments (*n* = 6; Duncan test at 95%).

Water consumption was similar to other crops, with a higher water supply (4774 m³ ha⁻¹) for 100% Rm and lower water supply (3695 m³ ha⁻¹) for 100% P_w_. But increased water application did not lead to increased yield. The superior performance of higher plant waste ratios in carrot might have been due to the improved soil structure, porosity and aeration, which are important for root growth and expansion. Our results are consistent with the reports that organic amendments increase root crop yield due to better soil structure and microbial activity^57,58^.

#### Comparative performance across crops

The control treatment consistently had the lowest yield and WP across all crops, showing the importance of organic amendments for crop yield and water productivity. 50% R_m_: 50% P_w_ was the best treatment for tomato and lettuce, but 25% R_m_: 75% P_w_ was the best treatment for carrot, suggesting crop-specific responses to the vermicompost blend.

WP was strongly negatively correlated with water applied, with balanced and higher proportions of plant waste recording an increase in WP with a decrease in water applied. This underscores the need for integrated nutrient and water management approaches to optimise crop production.

#### Implications for sustainable irrigation and nutrient management

This study shows integrated vermicompost treatments improve yield and water productivity under drip irrigation, and save water. The results align with the notion that balanced organic inputs enhance soil fertility, nutrient dynamics and water efficiency, key factors in sustainable agriculture.

### Growth response of vegetable crops to vermicompost feedstock composition

#### Tomato (*Solanum lycopersicum* L.)

The diameter varied from 120 cm in the control to 168 cm in the 50% rabbit manure + 50% plant/kitchen waste (50% R_m_: 50% P_w_) treatment, which is 40% greater than the control. The 25% R_m_: 75% P_w_ (159 cm) and 100% P_w_ (145 cm) treatments were intermediate, whereas 100% R_m_ (137 cm) had significantly smaller diameter than the mixed feedstocks. This suggests a feedstock combination that balances nutrient release from vermicompost, resulting in lateral plant development. The higher performance of mixed feedstocks compared to mono-feedstock vermicompost has been explained by complementary C: N ratios and higher micronutrients that promote cell division and growth^59^. Under fertigation, soluble humic and fulvic acids from mixed-feedstock. Vermicompost also activates auxin-dependent growth mechanisms, as reported by Canellas, Canellas^60^ for drip-irrigated vegetables (Table 6).

The highest root length was observed with 50% R_m_: 50% P_w_ (8.1 cm) and the lowest in the control (6.3 cm), followed by 100% R_m_ (7.2 cm) and 100% P_w_ (7.5 cm) which produced lower values than all mixed ratios. The incremental increase from 100% R_m_, to blended ratios, to the peak at 50:50 is explained by the combination of labile carbon (from plant/kitchen waste) and high nitrogen and phosphorus (from rabbit manure), which promote the rhizosphere microbial population to solubilise phosphorus and release root growth-promoting substances^59,61^. Improvement in the root system due to vermicompost under drip irrigation has been attributed to increased earthworm-derived growth promoters such as indole-3-acetic acid and cytokinins, which are active in the drip zone^60^(Table 6).

Leaf area index (LAI) was highest (4.0) in the 50% R_m_: 50% P_w_ treatment and lowest (2.9) in the control, followed by 100% P_w_ (3.0), which showed a near-minimum LAI. LAI, a key factor affecting light interception and photosynthesis, is responsive to different vermicompost feedstocks due to differences in nitrogen and potassium supply, which regulate leaf initiation and growth^3^. The lower LAI in 100% R_m_ (3.3) than in blended treatments confirms that rabbit manure, while rich in nitrogen, lacks some of the broad spectrum of organic acid fractions found in plant/kitchen waste vermicompost that are required for full leaf development^62^.

The largest fruit length and diameter (5.8 cm and 4.9 cm, respectively) were recorded in 50% R_m_: 50% P_w_ and smallest in the control (4.2 cm and 3.5 cm). The 100% P_w_ treatment produced smaller fruit (4.3 cm length, 3.7 cm diameter) than all blended treatments and 100% R_m_, suggesting the potassium and calcium requirements for tomato fruit growth are not fully satisfied by plant/kitchen waste vermicompost alone. Calcium, which controls cell wall structure and consequently fruit size, is also known to be higher in rabbit manure-based vermicomposts because of high calcium content of rabbit feed^61^. Fertigation also ensures the availability of these cations in the root zone, enhancing their absorption and directly contributing to fruit development, as reported by Boyacı, Kocięcka^63^ during water deficit (Table 6).

Chlorophyll (SPAD) showed a similar pattern as other growth attributes, with the highest value (45.3 SPAD) recorded in 50% R_m_: 50% P_w_ and the lowest (32.6 SPAD) in the control. The 100% P_w_ (34.0 SPAD) and 100% R_m_ (37.1 SPAD) treatments performed worse than the blends, supporting the idea that chlorophyll synthesis is supported by feedstock diversity. SPAD readings are a good indicator of leaf nitrogen content, and the blended vermicomposts probably offer a more balanced and long-term release of nitrogen that facilitates the production of chlorophyll precursors^3^.

The 25% R_m_: 75% P_w_ (39.4 SPAD) treatment was third best, and indicate diminishing returns as the plant/kitchen waste ratio increases beyond the optimal 50:50, likely due to lower N mineralization rates from higher C: N plant residues^59^.

#### Lettuce (*Lactuca sativa* L.)

The largest lettuce plant diameter (35 cm) was observed in 50% R_m_: 50% P_w_, while the smallest diameter (22 cm) occurred in the control, with a 59% increase. The 25% R_m_: 75% P_w_ (33 cm) and 75% R_m_: 25% P_w_ (32 cm) treatments were similar, but 100% P_w_ (30 cm) was significantly better than the control but worse than all mixes. The significantly lower performance of the control highlights the importance of organic nitrogen in promoting lettuce growth, which has a short growing cycle and low storage capacity^64^. The superior performance of the blends is consistent with reports of more gradual N release from mixed-feed vermicomposts, eliminating short-term N deficiencies that inhibit early leaf development in leafy crops^62^(Table 6).

Leaf nutrient concentrations were highest with 50% R_m_: 50% P_w_ (38 mg g^− 1^) and lowest with the control (24 mg g^− 1^), with 100% P_w_ (33 mg g^− 1^) and 100% R_m_ (32 mg g^− 1^) showing comparable intermediate values. The 50% R_m_: 50% P_w_ blend’s superior performance potentially stems from the combined nutrient content of the two feedstocks: rabbit manure vermicompost has high nitrogen and phosphorus loads, while plant/kitchen waste vermicompost supplies potassium, micronutrients and a wide range of humic substances that enhance root zone nutrient efficiency^60,61^. Drip fertigation ensures that nutrients are supplied directly to the root zone, reducing losses and enhancing foliar uptake, as reported by Somefun, Masasi^65^ in leafy vegetables (Table 6).

**Table 6:** Effect of different vermicompost media on vegetables characteristics.

Treatment	Tomato														
	Plant diameter (cm)	Root length (cm)			Leaf area index			Fruit length (cm)			Fruit diam. (cm)			Chlorophyll (SPAD)	
100% R_m_	137 ± 0.08b	7.2 ± 0.01b			3.3 ± 0.27bc			4.8 ± 0.33b			4 ± 0.13c			37.1 ± 0.23d	
75% R_m_ + 25% P_w_	152 ± 0.13d	7.6 ± 0.02c			3.8 ± 0.09a			5.5 ± 0.15a			4.6 ± 0.10b			42.6 ± 0.19b	
50% R_m_ + 50% P_w_	168 ± 0.06f	8.1 ± 0.11e			4 ± 0.07a			5.8 ± 0.18a			4.9 ± 0.25a			45.3 ± 0.33a	
25% R_m_ + 75% P_w_	159 ± 0.08e	7.8 ± 0.02d			3.5 ± 0.23b			5 ± 0.18b			4.3 ± 0.24b			39.4 ± 0.36c	
100% P_w_	145 ± 0.12c	7.5 ± 0.02c			3 ± 0.08 cd			4.3 ± 0.12c			3.7 ± 0.15 cd			34 ± 0.08e	
Control	120 ± 0.19a	6.3 ± 0.03a			2.9 ± 0.23d			4.2 ± 0.17c			3.5 ± 0.12d			32.6 ± 0.06f	

Treatment	Lettuce													
	Plant diameter (cm)		Root length (cm)			Root diameter (cm)			Leaf nutrient (mg g^− 1^)			Chlorophyll (SPAD)		
100% R_m_	30.8 ± 0.31c		9.7 ± 0.28 cd			1.4 ± 0.10ab			33.4 ± 0.74c			37.4 ± 0.42c		
75% R_m_ + 25% P_w_	33.6 ± 0.63b		10.6 ± 0.28ab			1.5 ± 0.23ab			36.5 ± 0.45b			40.8 ± 0.76b		
50% R_m_ + 50% P_w_	35 ± 0.14a		11 ± 0.13a			1.6 ± 0.15a			38 ± 0.34a			42.5 ± 0.48a		
25% R_m_ + 75% P_w_	32.9 ± 0.90b		10.3 ± 0.35bc			1.5 ± 0.16ab			35.7 ± 0.16b			39.9 ± 0.31b		
100% P_w_	29 ± 0.56d		9.1 ± 0.19d			1.3 ± 0.09ab			31.5 ± 0.53d			35.3 ± 0.55d		
Control	25.2 ± 0.18e		7.9 ± 0.19e			1.2 ± 0.21b			2.4 ± 0.18e			30.6 ± 0.46e		

Treatment	Carrot														
	Root length (cm)			Root diameter (cm)			Foliage diameter (cm)			Leaf nutrient (mg g^− 1^)			Chlorophyll (SPAD)		
100% R_m_	15 ± 0.06b			2.8 ± 0.01b			17.9 ± 0.18e			16.6 ± 0.11d			25.2 ± 0.27e		
75% R_m_ + 25% P_w_	17 ± 0.07c			3.2 ± 0.01c			21.5 ± 0.22d			20 ± 0.18c			30.3 ± 0.31d		
50% R_m_ + 50% P_w_	18 ± 0.09d			3.4 ± 0.02e			23 ± 0.10b			21.4 ± 0.38b			32.3 ± 0.34b		
25% R_m_ + 75% P_w_	19 ± 0.08e			3.6 ± 0.01f			24.2 ± 0.27a			22.5 ± 0.18a			34 ± 0.14a		
100% P_w_	17 ± 0.06c			3.3 ± 0.01d			22 ± 0.08c			20.5 ± 0.22c			30.9 ± 0.32c		
Control	13 ± 0.08a			2.5 ± 0.01a			16.5 ± 0.19f			15.3 ± 0.32e			23.1 ± 0.19f		

Means followed by different letters indicate significant differences between the treatments (*n* = 6; Duncan test at 95%).

Root diameter varied between 1.2 and 1.6 cm in the control and 50% R_m_: 50% P_w_ treatments, respectively, with all treatments statistically equivalent to each other except 100% P_w_ (1.3 cm). Despite the small numerical differences in lettuce root diameter, they are physiologically important because the formation of lateral roots determines the water and nutrient absorption capacity of shallow-rooted vegetables^66^. The small gain of 100% P_w_ over the control suggests that plant/kitchen waste alone does not contain sufficient readily mineralizable nitrogen to promote root development in the narrow wetting area of a drip irrigation system. The superiority of the 50% R_m_ + 50% P_w_ blend is in line with the reported stimulatory effects of amino acids and growth regulators from rabbit manure on root branching^59^.

The 50% R_m_: 50% P_w_ treatment exhibited the highest chlorophyll (42.5 SPAD) while the control had the lowest (30.6 SPAD). The 100% P_w_ (35.3 SPAD) treatment was placed fifth, with lower SPAD than the 100% R_m_ (37.4 SPAD) treatment, again showing that nitrogen from rabbit manure vermicompost is a more efficient source of chlorophyll synthesis than the carbon-rich amendments from plant/kitchen waste. The 25% R_m_: 75% P_w_ (39.9 SPAD) and 75% R_m_: 25% P_w_ (40.8 SPAD) treatments both had SPAD values above 39, suggesting that even low rabbit manure application rates can maintain high chlorophyll levels. These findings are in line with meta-analytical findings that vermicompost amendments consistently increase leaf chlorophyll concentration in leafy vegetables by increasing the availability of nitrogen, magnesium and iron^60,66^(Table 6).

#### Carrot (*Daucus carota* L.)

Carrot root length increased from 13 cm (control) to a maximum of 19 cm for 25% R_m_: 75% P_w_, the only crop and trait in this study where increasing the plant/kitchen waste ratio increased the optimum. The 50% R_m_: 50% P_w_ treatment (18 cm) followed, with 100% R_m_ (15 cm) being as good as the control; it is possible that the high proportion of rabbit manure may increase nitrogen uptake by the top, thus delaying storage root elongation. This is in line with antagonistic interactions between high leaf nitrogen and root sink strength in Apiaceous crops, where high leaf nitrogen inhibits assimilate translocation to the taproot^61^. The potassium-rich vermicompost obtained from plant/kitchen waste likely increases sucrose loading to phloem and supports taproot elongation through osmotic regulation, as found for root vegetable crops by Ma, Zhao^66^.

Root diameter was maximized under 25% R_m_: 75% P_w_ (3.6 cm) and minimized in the control (2.5 cm), with 50% R_m_: 50% P_w_ (3.4 cm) ranking second. The 100% R_m_ treatment (2.8 cm) had the smallest root among fertilized treatments, further confirming the trend observed for root length: excessive rabbit manure content is not conducive to carrot storage organ development. Potassium and boron, readily available from vermicompost and present in high concentrations in plant/kitchen waste amendments, are known to influence cambial activity and radial growth of root crops^59^. The specificity of drip fertigation in delivering these nutrients to the root zone has a more concentrated effect on radial growth, enhancing treatment differences (Table 6).

Foliage diameter varied from 16.5 cm (control) to 24.2 cm, the greatest among all carrot parameters in 25% R_m_: 75% P_w_. The 50% R_m_: 50% P_w_ (23.0 cm) and 100% P_w_ (22.0 cm) treatments were second and third, respectively, followed by the 100% R_m_ (17.9 cm) treatment, the poorest fertilized treatment. Carrot foliage diameter is a measure of plant health and its capacity to provide photoassimilates for taproot development^61^. The good performance of high plant/kitchen waste ratios for this trait is in line with the reported effects of humic acids and potassium on leaf area and canopy growth of root crops^60,66^.

Leaf nutrient content was highest under 25% R_m_: 75% P_w_ (22.5 mg g⁻¹) and lowest in the control (15.3 mg g⁻¹). The 50% R_m_: 50% P_w_ treatment (21.4 mg g⁻¹) and 100% P_w_ (20.5 mg g⁻¹) followed, while 100% R_m_ (16.6 mg g⁻¹) showed only marginal improvement over the control. The pattern differs from tomato and lettuce, where 100% R_m_ was superior to 100% P_w_, and suggests that for carrot, the broad spectrum of micronutrients (such as potassium, calcium, magnesium and trace elements) provided by plant/kitchen waste vermicompost plays a more critical role in leaf nutrient content than nitrogen-rich rabbit manure^3^. Our results indicate crop-specific vermicompost feedstock composition as a management tool in drip fertigated vegetable cultivation (Table 6).

Maximum chlorophyll (34.0 SPAD) was found in 25% R_m_: 75% P_w_, with the control at 23.1 SPAD, thereby confirming the major contribution of plant/kitchen waste vermicompost to carrot chlorophyll production. The 50% R_m_: 50% P_w_ treatment (32.3 SPAD) was the next highest, with 100% R_m_ (25.2 SPAD) only slightly better than the control, the greatest difference between the two single-feedstock treatments across the three crops. This reversal, with plant/kitchen waste being superior to rabbit manure for carrot chlorophyll content but not for tomato or lettuce, is probably due to the iron and manganese concentrations in the composted plant residues, both of which are essential cofactors in chlorophyll synthesis, and which are crucial for root crops in alkaline or calcareous soils^59,60^. The increasing SPAD values as P_w_ proportions increase for all carrot treatments (Table 6) highlights the importance of crop-specific feedstock blending strategies rather than “one size fits all” amendments for different vegetable crops.

### The impact of adding vermicomposting feedstock blending on soil properties

#### Effect of vermicomposting feedstock blending ratios on soil pH

**Fig. 6:**
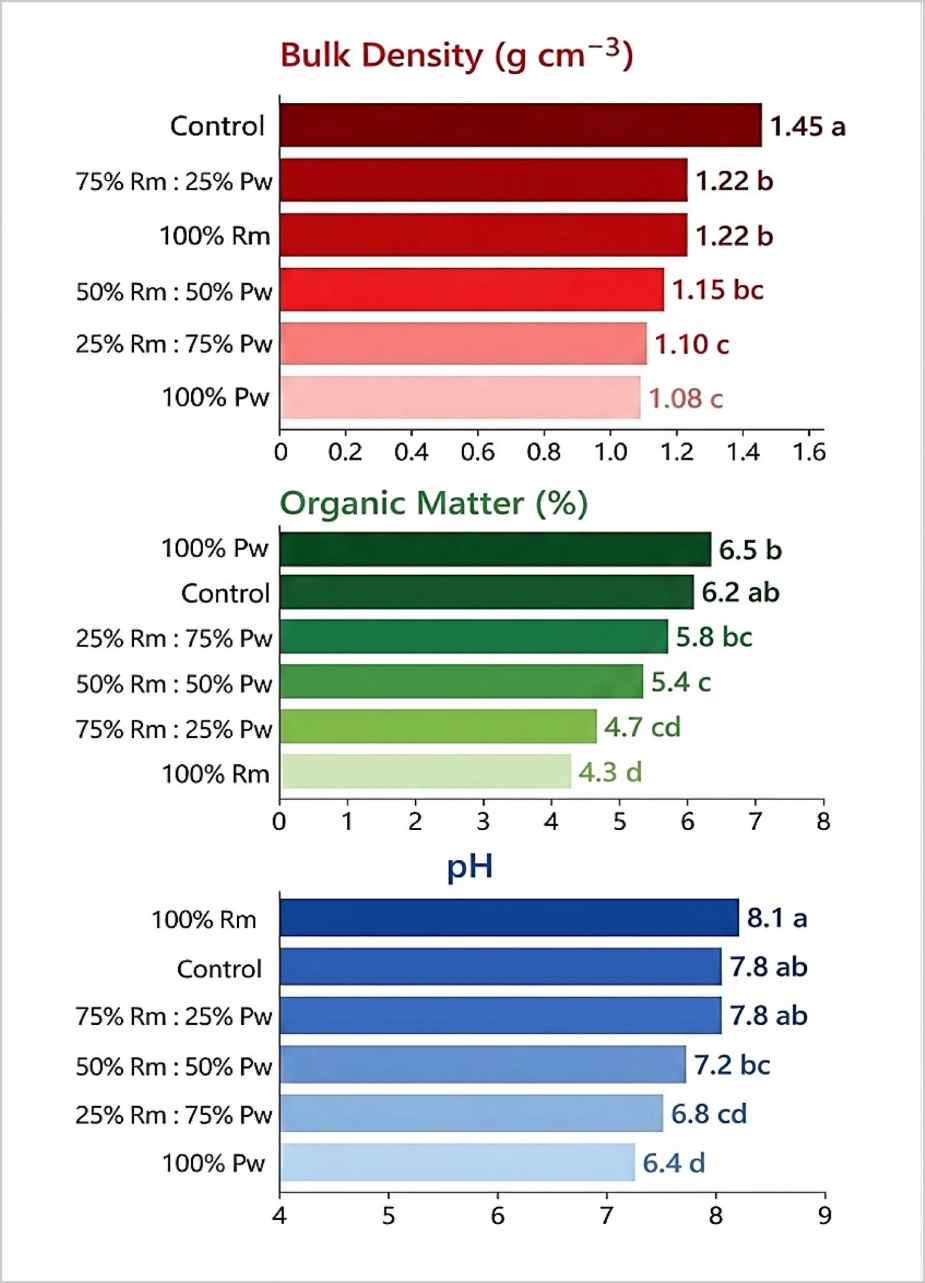
Vermicompost feedstock composition modulates soil pH, organic matter accumulation, and bulk density across rabbit manure–plant residue blending gradients. Different letters indicate a significant difference between treatments (*n* = 6; Duncan test at 95%).

Figure 6 show that soil pH significantly (*p* < 0.05) decreased with increasing ratios of plant/kitchen waste (P_w_) in the vermicompost feedstock, from 7.8 in 100% rabbit manure (R_m_) to 6.6 in 100% P_w_. Intermediate blends yielded pH values of 7.5 (75% R_m_: 25% P_w_), 7.2 (50% R_m_: 50% P_w_), and 6.9 (25% R_m_: 75% P_w_), while the unamended control recorded a pH of 7.1. The elevated pH of manure-rich feedstocks is due to the intrinsic alkalinity of rabbit manure, which contains carbonates and ammonia that resist acidification. Increasing P_w_ content caused a gradual release of short-chain organic acids, CO_2_ and humic compounds during the earthworm-driven degradation of lignocellulosic plant matter, thereby lowering pH via proton generation and complexation reactions.

The pH values across all treatments fell within the Agronomically recommended range of 6.5–7.8 for most crop plants, suggesting that the blending ratios tested did not result in phytotoxic acidification^67–69^.

#### Effect of vermicomposting feedstock blending ratios on soil organic matter

Soil organic matter (OM) content increased significantly (*p* < 0.05) with rising P_w_ proportions, following the order: 100% P_w_ (6.2%) > 25% R_m_: 75% P_w_ (5.9%) > 50% R_m_: 50% P_w_ (5.7%) > 75% R_m_: 25% P_w_ (5.2%) > 100% R_m_ (4.8%) > Control (2.4%). The control treatment had the lowest OM (2.4%) which illustrates the loss of soil carbon under continuous cropping without organic amendment. The OM content in all vermicompost treatments surpassed the threshold level of 4% reported in the literature as the minimum level required for optimal soil biology and structure (Fig. 6). The greater OM build-up in P_w_-rich treatments is mechanistically explained by the plant residues’ higher lignin and cellulose content, which slows down mineralization and releases stable humic carbon into the soil carbon pool upon vermicompost application. The 158% OM increase from the control (2.4%) to the 100% P_w_ treatment (6.2%) highlights the ability of plant-waste-rich vermicomposts to quickly restore the soil organic carbon pool, which is essential for soil health in intensively cropped systems^67,69,70^.

#### Effect of vermicomposting feedstock blending ratios on soil bulk density

The bulk density (BD) of soil was significantly (*p* < 0.05) lower with all the vermicompost treatments compared to the control (1.45 g cm^− 3^). The lowest BD was recorded in the 100% P_w_ treatment (1.19 g cm^− 3^), representing a 17.9% reduction compared with the control, followed by 25% R_m_: 75% P_w_ (1.21 g cm^− 3^), 50% R_m_: 50% P_w_ (1.24 g cm^− 3^), 75% R_m_: 25% P_w_ (1.28 g cm^− 3^), and 100% R_m_ (1.31 g cm^− 3^).

The gradual reduction in BD with increasing proportion of Pw is due to the higher fiber content of the plant-waste derived vermicomposts that provide a more stable structural framework, especially from partially decomposed lignin and cellulose, to the soil, increasing its total pore space and decreasing particle packing. Notably, all vermicompost treatments produced BDs below 1.35 g cm^− 3^, the generally accepted threshold at which root and water movement are severely constrained in most soils. By contrast, the control BD (1.45 g cm^− 3^) was above this threshold, suggesting a structurally degraded soil with poor aeration and drainage that would restrict crop production^71–73^ (Fig. 6).

### Impact of vermicomposting sources on soil cation exchange capacity (CEC), water holding capacity (WHC), and infiltration rate (IR)

**Fig. 7:**
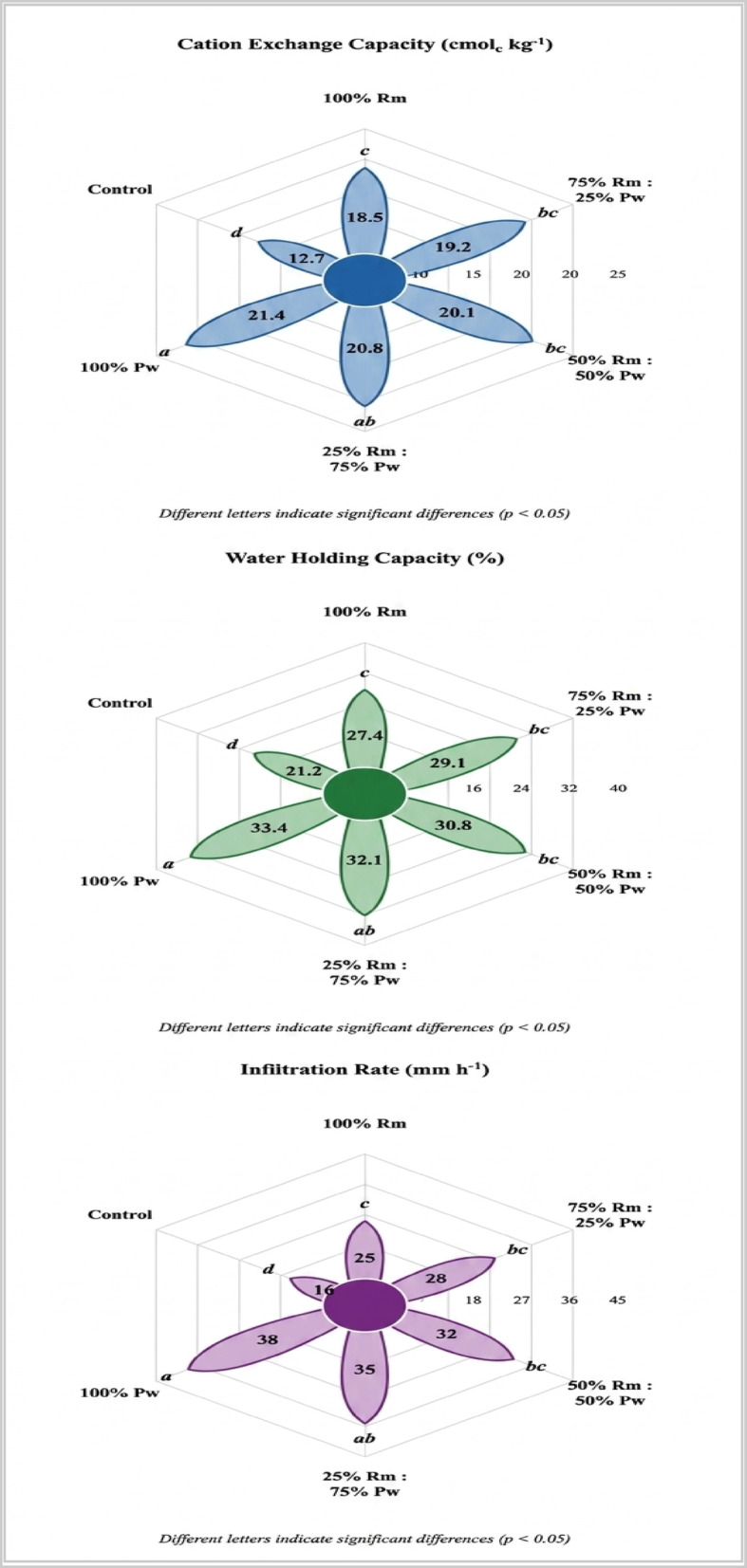
Effects of rabbit manure and plant/kitchen waste Ratios on soil physicochemical properties: cation exchange capacity, water holding capacity, and infiltration rate.

The application of various vermicomposting sources significantly influenced key soil properties, namely Cation Exchange Capacity (CEC), Water Holding Capacity (WHC), and Infiltration Rate (IR). A clear trend emerged where increasing proportions of plant/kitchen waste (P_w_) in the vermicompost mixture, relative to rabbit manure (R_m_), generally led to enhanced soil characteristics. The control group, which received no vermicompost, consistently exhibited the lowest values across all measured parameters, underscoring the beneficial impact of vermicompost application (Fig. 7).

Cation Exchange Capacity (CEC), a crucial indicator of soil fertility and nutrient retention, showed a progressive increase with higher percentages of plant waste in the vermicompost. The lowest CEC value among the treated soils was observed with 100% R_m_ vermicompost at 18.5 cmol_c_ kg^-1^. This value steadily rose to 19.2 cmol_c_ kg^-1^ for 75% R_m_ + 25% P_w_, 20.1 cmol_c_ kg^-1^ for 50% R_m_ : 50% P_w_, 20.8 cmol_c_ kg^-1^ for 25% R_m_ + 75% P_w_, and peaked at 21.4 cmol_c_ kg^-1^ for 100% P_w_ vermicompost. All vermicompost treatments demonstrated superior CEC compared to the control, which recorded a significantly lower value of 12.7 cmol_c_ kg^-1^. This enhancement in CEC can be attributed to the increased organic matter content and the formation of humic substances during vermicomposting, particularly from plant-based materials, which possess a higher number of negatively charged sites capable of adsorbing cations^74^.

Water Holding Capacity (WHC), vital for plant water availability and drought resilience, followed a similar pattern of improvement. The 100% R_m_ treatment resulted in a WHC of 27.4%, which increased with the incorporation of plant waste: 29.1% for 75% R_m_ + 25% P_w_, 30.8% for 50% R_m_ + 50% P_w_, 32.1% for 25% R_m_ + 75% P_w_, and reaching its maximum at 33.4% with 100% P_w_ vermicompost (Fig. 7). The control soil exhibited a substantially lower WHC of 21.2%. The observed increase in WHC is consistent with findings from other studies, which report that vermicompost improves soil aggregation and pore space distribution, thereby enhancing its capacity to retain water^67^. The higher fibrous content and structural stability imparted by plant waste-derived vermicompost likely contributed to this trend.

Soil infiltration rate (IR), indicative of water movement into the soil and aeration, also showed a positive correlation with the proportion of plant waste in the vermicompost. The IR for 100% R_m_ was 25 mm h^-1^, increasing to 28 mm h^-1^ for 75% R_m_ + 25% P_w_, 32 mm h^-1^ for 50% R_m_ + 50% P_w_, 35 mm h^-1^ for 25% R_m_ + 75% P_w_, and reaching the highest rate of 38 mm h^-1^ with 100% P_w_ vermicompost (Fig. 7). The control soil had a markedly lower IR of 16 mm h^-1^. The improved infiltration rates are likely due to the enhanced soil structure, increased macroporosity, and reduced bulk density facilitated by vermicompost, particularly those rich in plant residues^75^. These physical improvements allow for better water penetration and reduced runoff (Fig. 7).

The findings of this study align with and further support a growing body of literature on the beneficial effects of vermicompost on soil properties. Recent research consistently highlights vermicompost as an effective organic amendment for improving soil fertility and physical characteristics^76^.

For instance, studies have shown that vermicompost application leads to significant increases in CEC, often attributed to the high content of humic acids and organic colloids that provide abundant negative charges for cation adsorption^74^. This is particularly relevant for soils with low inherent fertility, where vermicompost can substantially boost nutrient retention capacity. The observed trend of increasing CEC with higher plant waste content in vermicompost is consistent with the understanding that plant-derived organic matter often has a more stable and complex structure, contributing to long-term CEC improvements^77^.

Similarly, the enhancements in WHC observed in this study are well-documented in the literature. Vermicompost improves soil aggregation, creating a more stable soil structure with a greater proportion of micropores and macropores that can hold water more effectively^67,78^. A study by Castellini, Bondì^67^ specifically noted that vermicompost addition improved soil water holding capacity, with effects varying depending on soil texture, but generally showing positive impacts on plant available water capacity. Our results, showing a maximum WHC of 33.4% with 100% P_w_ vermicompost compared to 21.2% in the control, are in line with these reported benefits. Regarding infiltration rate, the literature confirms that vermicompost application can significantly enhance water movement into the soil by improving soil structure, reducing bulk density, and increasing porosity^75,79^.

The higher infiltration rates observed with increasing plant waste content suggest that these materials contribute to the formation of stable aggregates, which are crucial for maintaining good soil aeration and water permeability. This is a critical aspect for preventing surface runoff and promoting efficient water utilization in agricultural systems.

In conclusion, the present study demonstrates that vermicompost, especially formulations rich in plant waste, is a potent soil amendment capable of significantly improving CEC, WHC, and IR. These findings are strongly supported by recent academic literature, reinforcing the role of vermicomposting as a sustainable practice for enhancing soil health and productivity.

### Effects of organic amendment ratios on soil biological activity, microbial biomass, and faunal dynamics

Soil biological indicators were substantially affected by the organic amendments (R_m_ and P_w_) with consistent trends across the gradient of treatments (Table 7). As P_w_ content increased, there were significant (*p* ≤ 0.05) increases in microbial, enzymatic and faunal parameters compared to the control and R_m_-only treatments. Our results align with recent research showing organic amendments improve soil biological functions and ecosystem processes^80,81^.

Microbial biomass carbon (MBC) grew significantly from 235 mg kg⁻¹ in the control treatment to 612 mg kg⁻¹ with 100% P_w_. Combined treatments exhibited gradual increases with increasing P_w_. This suggests that P_w_ offers more easily available carbon sources, promoting microbial activity. This is in line with recent meta-analyses that showed organic amendments significantly increased microbial biomass, as a result of enhanced carbon and nutrients supply^75^. Long-term studies also indicate that such increases in MBC reflect improved soil fertility and carbon cycling^81^.

Similarly, dehydrogenase activity (DHA) increased from 87 to 238 µg TPF g⁻¹ day⁻¹. This indicates improved microbial oxidation and metabolism. The high correlation between MBC and DHA indicates that the increased microbial population under P_w_ treatments is active. Recent studies have also shown similar increases in DHA due to organic amendment, highlighting its sensitivity to soil biological processes^82,83^.

Earthworm numbers significantly increased from 41 individuals’ m⁻² in the control to 134 individuals’ m⁻² under 100% P_w_. This increase reflects the improved soil habitat, probably because of the higher organic matter, better soil structure and food for soil animals. Organic matter is well known to stimulate soil macrofauna through aggregating, aerating, and retaining moisture. Our results align with recent research showing the role of organic amendments in improving soil biological properties and ecosystem functions^84^.

Basal respiration rates rose from12 to 32 mg CO₂ kg^-1^ soil day^-1^, reflecting increased microbial respiration and carbon mineralisation. The respiratory response in P_w_-dominated treatments indicates higher decomposition of easily available organic matter. This is consistent with global meta-analyses that reveal organic amendments enhance microbial respiration and carbon cycling by supplying labile substrates^85^. But although higher respiration rates indicate active microbial activity, it also suggests a balance between carbon mineralization and carbon storage.

The fungal-to-bacterial (F: B) ratio rose from 0.08 in the control to 0.25 with 100% P_w_, suggesting a fungal dominance. This can lead to better soil aggregation, increased carbon efficiency and ecosystem resilience. Recent research has demonstrated that organic amendments can shift microbial communities towards increased fungal dominance, which is important for soil aggregation and carbon sequestration^81,86^.

**Table 7:** Influence of rabbit manure/plant and kitchen amendment ratios on soil microbial biomass, enzyme activity, and earthworm abundance.

Parameter	Microbial biomass carbon (mg kg^− 1^)	Dehydrogenase activity (µg TPF g^− 1^ day^− 1^)	Earthworm population (No. m^− 2^)	Basal respiration (mg CO_2_ kg^− 1^ soil day^− 1^)	Fungal/bacterial ratio
100% R_m_	485 ± 0.11 b	178 ± 0.02 b	82 ± 0.07 b	21 ± 0.07 b	0.12 ± 0.02 b
75% R_m_ : 25% P_w_	524 ± 0.17 c	192 ± 0.06 c	95 ± 0.08 c	24 ± 0.07 c	0.15 ± 0.02 c
50% R_m_ : 50% P_w_	567 ± 0.06 d	215 ± 0.07 d	112 ± 0.14 d	28 ± 0.03 d	0.18 ± 0.01 d
25% R_m_ : 75% P_w_	594 ± 0.01 e	226 ± 0.07 e	121 ± 0.08 e	30 ± 0.11 e	0.21 ± 0.01 e
100% P_w_	612 ± 0.15 f	238 ± 0.05 f	134 ± 0.17 f	32 ± 0.15 f	0.25 ± 0.01 f
Control	235 ± 0.08 a	87 ± 0.02 a	41 ± 0.08 a	12 ± 0.06 a	0.08 ± 0.01 a

Means followed by different letters indicate significant differences between the treatments (*n* = 6; Duncan test at 95%).

The improvement in all the parameters indicates that P_w_ is a valuable organic amendment to enhance soil biological quality. The effectiveness of the 100% P_w_ treatment, as well as the significant responses in the mixed amendments (especially 50:50 and 25:75), indicate both additive and synergistic interactions of organic amendments.

These findings are well supported by recent studies that suggest the quality and composition of organic amendments are crucial in controlling soil microbial dynamics, enzyme activity and ecosystem functioning^81,83^. The results demonstrate that higher proportions of P_w_ significantly improve soil biological quality by enhancing microbial biomass, enzyme activity, respiration, and soil fauna, and inducing favorable microbial community shifts. This highlights the critical role of organic amendment management for enhancing soil health and sustainability.

### Economic feasibility of irrigation systems with vermicompost feedstock composition

#### Fixed and variable costs

Fixed costs (FC) were constant for all three crops (US$ 2647.47 ha⁻¹ y⁻¹), mainly comprising seasonal land rent (US$ 1812.30 ha⁻¹) and labor costs (US$ 571.10 ha⁻¹). The vermicompost reactor was minimal at US$ 7.61 ha⁻¹, and confirms its low investment and affordability for small holders^87^.

Variable costs (VC) varied substantially by crop: tomato (US$ 1,890.33 ha⁻¹ y⁻¹), lettuce (US$ 1,238.79 ha⁻¹ y⁻¹), and carrot (US$ 1,065.72 ha⁻¹ y⁻¹). Vermicompost was the largest VC (US$ 815.54, US$ 679.61, and US$ 611.65 ha⁻¹ y^− 1^, respectively), accounting for 43–57% of VC for all three crops. Seedling VC was highest for tomato (US$ 555.02 ha⁻¹), due to transplanting, while irrigation VC was lowest for lettuce (US$ 57.11 ha⁻¹ y⁻¹), reflecting its lower water demand^63^ (Table 8).

#### Total revenue, yield, and net farm income

Tomato led in yield (102 ton ha⁻¹), well above the global average yield of ~ 34 ton ha⁻¹^65^, followed by carrot (57 ton ha⁻¹) and lettuce (45 ton ha⁻¹) (Table 7). This is due to the combined effect of precision drip irrigation and vermicompost which has been reported to increase water use efficiency and marketable yield^3,63^. At market prices of US$ 300, US$ 285, and US$ 350 ton⁻¹, total revenues were US$ 30,600, US$ 12,825, and US$ 19,950 ha⁻¹ y⁻¹ for tomato, lettuce, and carrot, respectively. Net Farm Income (NFI = TR − TC) was US$ 25,917.51 ha⁻¹ y⁻¹ for tomato, US$ 16,092.12 ha⁻¹ y⁻¹ for carrot, and US$ 8,794.05 ha⁻¹ y⁻¹ for lettuce (Table 7). This is much higher than the NFI reported for conventional drip-irrigated tomato farming (US$ 3,000–7,000 ha⁻¹ y⁻¹^88^, due to both the positive effect of vermicompost on yield and premium prices for low-chemical production. For carrot and lettuce, results are in line with meta-analyses showing that vermicompost amendments benefit marketable yield and soil properties in vegetables^64,66^.

#### Circular economy implications

The integrated vermicompost reactor in the production system exemplifies circular economy (CE) principles by transforming farm organic wastes into a valuable biofertilizer product, recycling farm nutrients, and offsetting synthetic nitrogen (N) fertilizer use (up to 20%)^87^. Its low fixed cost (US$ 7.61 ha⁻¹) facilitates CE adoption. The system offers further revenue opportunities from the sale of earthworm biomass US$ 5–35 per pound in some markets^89^ and waste management savings when sourced from nearby municipal streams, turning the variable cost of vermicompost into a cost offset. The global vermicompost market, estimated at ~ US$ 102 billion in 2024 and US$ 335 billion by 2032 (CAGR 16%), further justifies such systems’ strategic positioning in an increasingly organic market and regulatory environment^90^. Its pairing with drip irrigation further enhances CE by facilitating fertigation and reducing environmental nutrient losses, potentially leading to a 10% cost reduction^63,65,91^.

**Table 8:** Economic feasibility analysis for calculation of the net income of irrigation systems with vermicompost feedstock composition in vegetables production.

Item		Costs (US$ ha^− 1^ y^− 1^)			
		Tomato	Lettuce	Carrot	
Fixed costs (FC)	Administrative expenses	571.1	571.1	571.1	
	Vermicompost reactor	7.61	7.61	7.61	
	Irrigation system	181.23	181.23	181.23	
	Annual consumption 10% (without land rent)	75.23	75.23	75.23	
	Rent (on season)	1812.3	1812.3	1812.3	
Total FC		2647.47	2647.47	2647.47	
Variable costs (VC)	Irrigation	85.66	57.11	85.66	
	Seedlings	555.02	226.54	101.94	
	Vermicompost	815.54	679.61	611.65	
	Weed control	113.27	67.96	72.49	
	Harvesting	362.46	226.54	271.85	
	Maintenance (2.2–7.4% FC)	139.608	139.608	139.608	
Total VC		2071.558	1397.37	1283.2	
Total revenue (TR)	Yield (ton ha^− 1^)	102.00	45.00	57.00	
	Price (300, 285, and 350 US$ ton^− 1^)	30600.00	12825.00	19950.00	
Net farm income (NFI) = TR - TC		25880.97	8780.16	16019.3	

## Conclusions

Through this research, it is proven that no specific proportion of Rm can be combined with P_w_ to produce quality vermicompost and hence there is no one size fits all solution to the problem. Specifically, R_m_ shortens the composting process to 75 days with a peak temperature of 68 °C, while P_w_ extends it to 120 days; a blend of 50:50, however, does the longest thermophilic (25 days), which results in the most effective destruction of pathogens and stability of the compost.

The physical and chemical properties of the soil are improved significantly in most cases of P_w_-dominated blends (increase in organic matter, porosity, water holding capacity (WHC), cation exchange capacity (CEC), decrease in bulk density below 1.35 g cm^− 3^, pH and electrical conductivity (EC); while the 50:50 blend yields the most optimum agronomic profile (pH 7.4, EC 3.7 dS m^− 1^, C/N ratio 21) for the mature, non-saline compost.

In the nutritional sense, R_m_-rich blends provide the highest levels of macronutrients (N, P, K, Ca) and most micronutrients (Fe, Mn, Zn, Cu) except Pw, which specifically raises the amount of boron. Remarkably, the 50:50 mix retains around 71% of the N and 81% of the Fe as compared to pure Rm, suggesting that partial replacement does not significantly affect fertility.

In terms of crop productivity, all treatments significantly out yield the unfertilized control, with the 50:50 Rm: Pw ratio being best for tomato (10.2 kg m^− 2^) and lettuce (4.5 kg m^− 2^), while the 25:75 Rm: Pw ratio is best for carrot (5.7 kg m^− 2^) owing to improved soil structure for root growth. Therefore, the 50:50 ratios of fruit and leafy vegetables is recommended, the 25:75 ratio for root crops, and the 75–100% P_w_ formulation for sandy or degraded soils, and it is recommended to plant on infertile soils with a mixture that is dominated by P_w_, then gradually introduce P_w_ into future planting seasons.

Nevertheless, this study highlights several areas of research that still need to be explored to better understand the effects of the different vermicompost blends. These include the long-term cumulative and residual effects of these different vermicompost blends on soil microbial community dynamics across multiple cropping cycles; the exact biochemical mechanisms responsible for the distinct boron enrichment in the Pw dominated treatments would benefit from deeper investigation at the molecular level; and future research should perform economic feasibility and life-cycle assessments (LCA) to connect the experimental results with the scalability to commercial scale.

## Limitations and future directions

Future research in this area should consider field verification of multiple applications in subsequent seasons of vermicompost for carbon sequestration and yield stability under field conditions beyond the greenhouse level of this study, GHG emissions during vermicomposting for different blending ratios to complete the environmental life cycle assessment of the process, and climate-smart production.

1. Crop and soil responses were evaluated in a controlled greenhouse environment rather than in an open field environment. Future work should evaluate the 50:50 Rm: Pw formulation in the field over several growing seasons to ensure yield stability and residual effect ts on the soil.
2. The vermicomposting process was carried out in 27 L bench-scale glass reactors and may not completely reflect thermal and aeration dynamics of pilot or industrial scale of composting. Recommendation: Scale-up trials are advised using pilot or commercial scales reactors to ensure the process consistency before field level.
3. Only one sandy loam soil was assessed for crop and soil quality. Solution: Tests of a variety of soil textures (clay, silty) would enable a generalization of the optimum Rm: Pw ratios recommended in this study.
4. The amount of GHG emitted during the vermicomposting process was not measured. Answer: As mentioned in our Conclusion, future research is needed to include GHG monitoring and a complete life-cycle assessment of the process to consider the overall environmental impact.

## Data Availability

All data included in the research will be made available upon request through Prof. Dr. Mohamed Abuarab (mohamed.aboarab@agr.cuedu.eg), while the data from previous studies and research was obtained through the Cairo University platform, which provides research information on a regular basis.

## Abbreviations

AAS: Atomic absorption spectroscopy
AOAC: Association of official analytical chemists
BD: Bulk density
C/N: Carbon-to-nitrogen ratio
Ca: Calcium
CEC: Cation exchange capacity
CRD: Completely randomized design
DHA: Dehydrogenase activity
DTPA: Diethylenetriaminepentaacetic acid
EC: Electrical conductivity
ET_O_: Reference evapotranspiration
FC: Fixed costs
GC: Ground cover
GHG: Greenhouse gas
GI: Germination index
LAI: Leaf area index
LR: Leaching requirement
LSD: Least significant difference
MBC: Microbial biomass carbon
NFI: Net farm income
OM: Organic matter
PLFA: Phospholipid fatty acid
P_w_: Plant/Kitchen waste
R_m_: Rabbit manure
SPAD: Chlorophyll meter reading
TPF: Triphenyl formazan
TR: Total revenue
WHC: Water holding capacity
WP: Water productivity
WRC: Water retention capacity
